# Evaluation of the implementation of an integrated primary care network for prevention and management of cardiometabolic risk in Montréal

**DOI:** 10.1186/1471-2296-12-126

**Published:** 2011-11-10

**Authors:** Sylvie Provost, Raynald Pineault, Pierre Tousignant, Marjolaine Hamel, Roxane Borgès Da Silva

**Affiliations:** 1 Direction de santé publique de l'Agence de la santé et des services sociaux de Montréal, Canada; 2 Institut national de santé publique du Québec, Canada; 3 Centre de recherche du Centre hospitalier de l'Université de Montréal, Canada

## Abstract

**Background:**

The goal of this project is to evaluate the implementation of an integrated and interdisciplinary program for prevention and management of cardiometabolic risk (PCMR). The intervention is based on the Chronic Care Model. The study will evaluate the implementation of the PCMR in 6 of the 12 health and social services centres (CSSS) in Montréal, and the effects of the PCMR on patients and the practice of their primary care physicians up to 40 months following implementation, as well as the sustainability of the program. Objectives are: 1-to evaluate the effects of the PCMR and their persistence on patients registered in the program and the practice of their primary care physicians, by implementation site and degree of exposure to the program; 2-to assess the degree of implementation of PCMR in each CSSS territory and identify related contextual factors; 3-to establish the relationships between the effects observed, the degree of PCMR implementation and the related contextual factors; 4-to assess the impact of the PCMR on strengthening local services networks.

**Methods/Design:**

The evaluation will use a mixed design that includes two complementary research strategies. The first strategy is similar to a quasi-experimental "before-after" design, based on a quantitative approach; it will look at the program's effects and their variations among the six territories. The effects analysis will use data from a clinical database and from questionnaires completed by participating patients and physicians. Over 3000 patients will be recruited. The second strategy corresponds to a multiple case study approach, where each of the six CSSS constitutes a case. With this strategy, qualitative methods will set out the context of implementation using data from semi-structured interviews with program managers. The quantitative data will be analyzed using linear or multilevel models complemented with an interpretive approach to qualitative data analysis.

**Discussion:**

Our study will identify contextual factors associated with the effectiveness, successful implementation and sustainability of such a program. The contextual information will enable us to extrapolate our results to other contexts with similar conditions.

**Trial registration:**

ClinicalTrials.gov: NCT01326130

## Background

Evaluation of effective and sustainable interventions in chronic disease management and of their implementation in various contexts is essential to guide such initiatives in Canada. One initiative that seems promising but presents a challenge to be addressed by decision makers at all levels is the creation of primary care-centred integrated networks that foster and are congruent with the development of local services networks. Our study findings will support efforts to institute local integrated services networks throughout Canada.

The Agence de la santé et des services sociaux de Montréal (ASSSM) has commissioned our team to help evaluate the implementation of an integrated and interdisciplinary cardiometabolic risk (diabetes and hypertension) management and prevention program. The intervention is based on the Chronic Care Model (CCM), in a context where integrated services networks are created. Although shorter-term effects can be detected and the implementation process documented over a short time period, longer-term effects on patients registered in the program and on the practices of participating physicians, as well as the sustainability of implemented actions, require a long enough observation period to take place. The project is scheduled to last 40 months.

### Some epidemiological data

The growing burden of chronic diseases such as diabetes and their impact on the health system are now obvious [1]. Population ageing and changes in lifestyles, especially in terms of diet and sedentarity, are markedly responsible for this increased burden. In 2006-07, nearly 6 million Canadians aged 20 years and older were living with diagnosed hypertension (24.0% of women and 21.3% of men) [2]; over 2 million Canadians aged 20 years and older, or 8.0% of the population, were diagnosed with diabetes (7.5% of women and 8.6% of men) [3]. The age-adjusted prevalence of hypertension in Canada rose 52% from 1998-99 to 2006-07; for diabetes, it increased by 21% between 2002-03 and 2006-07. In 2006-07, 5.1% of Canadians aged 20 years and older were living with both diabetes and hypertension; 22.7% of adults with hypertension also had diabetes, and 62.8% of adults with diabetes also had hypertension [2]. These data demonstrate the relevance and significance of considering the two diseases together. In addition, both have common risk factors and complications.

### Recommendations - Hypertension and diabetes

The most recent clinical practice guidelines for diabetes [4,5] and hypertension [6] give evidence-based recommendations pertaining to follow-up of people with diabetes or hypertension. Optimization of glycemic control is fundamental to adequate patient management. Glycated hemoglobin (HbA1c) can reliably assess average glycemia over the past 3 to 4 months [7] and is a good indicator of treatment efficacy [5]. In most patients with diabetes, treatment must achieve a median HbA1c value ≤ 7.0% to reduce the risks of microvascular [8] and macrovascular [9] complications. Hypertension guidelines set the target level for blood pressure at 140/90 mm/Hg; the target level for people with diabetes is 130/80 mm/Hg.

For optimal glycemia and hypertension control, the guidelines target lifestyle modification (diet, physical activity and smoking cessation) and risk factor management (dyslipidemia and obesity), in addition to pharmaceutical treatment. Support for disease self-management is also an integral part of an effective strategy for chronic disease management. Individuals with chronic diseases must cope on a daily basis with the symptoms and consequences of the disease, and are mostly responsible for enacting appropriate strategies (e.g. lifestyle modifications, measurement of blood glucose levels or of blood pressure at home) that will enable them to maintain a satisfactory quality of life [10]. Self-management is considered to be an effective way to narrow the gap between the needs of people with chronic diseases and the capacity of the health system to respond to those needs.

Moreover, the guidelines indicate that although short clinical interventions increase the likelihood that patients adopt and maintain healthy lifestyle habits, interdisciplinary care approaches are more effective [12,13]. Care must be provided systematically and supported by organizational interventions (e.g. computerized databases, decision trees, patient and team member recall systems). It is essential that the family physician, who ensures continuity of care, and the patient care team coordinate care and share clinical information. In a care model based on collaboration, the care team should be given appropriate support and training (e.g. through the participation of a specialist). Studies of chronic care management programs indicate that the Chronic Care Model (CCM) developed by Wagner et al. [14] contains the necessary elements to improve processes of care as well as the health and quality of life of patients with chronic diseases [15,16].

### The Chronic Care Model

A number of care models have been developed to improve chronic disease management. The Chronic Care Model [14] is the one most often used. It is based on integrating services at different levels of the health system, and revolves around six interrelated elements of care organization: 1) delivery system design (coordinated and integrated multidisciplinary teams with systematic sharing of clinical information); 2) self-management support for patients; 3) decision support (interventions designed to improve providers' knowledge and skills); 4) development of a clinical information system (computerized patient registry and computerized evaluation and follow-up tools); 5) use of community resources; and 6) healthcare organization centred on chronic illness. The CCM posits that better outcomes are likely to result from interactions between a proactive team of well-prepared professionals and active, informed patients [17].

Several studies, particularly of diabetes patients, have shown that interventions based on CCM components improve processes and outcomes, and reduce costs and service utilization among patients with chronic illnesses [18]. However, most of these studies focus on management of single morbidities, from a disease rather than case management perspective [19]. Yet, people with chronic diseases often present multiple comorbidities. To provide effective care, the CCM should be oriented toward case management to take into account multiple morbidities [19,20].

Moreover, the literature indicates that primary care services are best suited to provide chronic disease management, although few studies have analyzed factors facilitating implementation of the CCM in primary care settings [19,20]. A number of factors that facilitate or hinder implementation of the CCM in various contexts have been reported in the literature [14,21-24]. A lack of human and financial resources and the absence of appropriate clinical information systems are the most common barriers reported. An organizational culture that fosters change, the quality of managerial leadership, management strategies that frame organizational changes (objectives clearly defined, organizational strategic plan developed, and trusting relationships among stakeholders), physician support, vision and values related to changing practices, medical leadership that supports innovation, and the presence of championing physicians are all significant to successful implementation.

### Implementation of local health networks

Chronic illness management requires better integration of services designed for individuals affected with such diseases. It is in part to meet these needs that, in 2004, 95 health and social services centres (CSSS) were created in Québec, 12 of which being in Montréal. The Centres resulted from the merging of local community health centres, long-term care facilities and, in most cases, a hospital. The goal of this new entity is to bring health services closer to the population, make them more accessible and improve their coordination and continuity in each territory. CSSS have also been given the mandate to set up local services networks by encouraging collaboration among organizations and partners in their territories, more particularly primary care services [25].

Several strategies can be adopted to create local services networks, one of which is to set up integrated services networks based on disease or population target groups. Studies have shown that implementation of health networks is facilitated by the development of initiatives that foster integration of services for targeted groups of patients with chronic illnesses or with distinct demographic characteristics [26-29]. In addition, clinical integration is a prerequisite to systemic integration [30]. In this sense, by favouring the establishment of integrated services networks, the CCM is a first-rate instrument to create local services networks and to consolidate their implementation.

### Initiatives in Montréal and implementation of a cardiometabolic risk program

The Agence de la santé et des services sociaux de Montréal (ASSSM) has undertaken activities designed to improve management of chronic illnesses and inspired by the Chronic Care Model. In 2006, following a strategic initiative launched by the ASSSM, the CSSS Sud-Ouest-Verdun set up a diabetes referral centre. Since then, the program has become a model for the other CSSS in Montréal, who have shown an interest in developing similar programs in their territories. Due to the frequent association between diabetes and hypertension, the ASSSM has recommended adding the latter to the program. The ASSSM proposes to continue to implement the integrated and interdisciplinary program for prevention and management of cardiometabolic risks (PCMR) first in six CSSS territories in Montréal (including the CSSS Sud-Ouest-Verdun), and then eventually in all CSSS in Montréal. Each CSSS will implement the program with the perspective of having an integrated care network in its territory, taking into account its specific organizational context. The program aims to improve diabetes and hypertension control among the target clientele (early detection, control of risk factors, optimal non-pharmacological and pharmacological management); enhance the quality of life of people with dysglycemia and/or hypertension; consolidate primary care case management and optimize use of specialized services; and strengthen links with various partners to offer integrated services in the CSSS territory. Targeted clienteles are adults with marginal fasting glycemia, or glucose intolerance, or diabetes treated with diet only, or diabetes treated with monotherapy, or diabetes treated with more than one medication if HbA1c ≤ 8.0%; as well as adults with hypertension with BP in the doctor's office ≥ 140/90 mm Hg (if diabetic, with BP ≥ 130/80).

The PCMR anticipates implementing a **clinical process **in each CSSS that aims, over a two-year period, to change lifestyle habits, control biological indicators, prevent complications and support patient self-management. Clinical interventions include group educational sessions given by the nutritionist, nurse, kinesiologist, pharmacist and psychosocial worker; individual follow-up by the nutritionist and the nurse; participation in a physical activity program; monitoring of biological parameters; and referral to specialists, as needed. In each CSSS, follow-up and educational activities are provided by a cardiometabolic risk education centre's interdisciplinary team, complementarily with medical follow-up provided by the primary care physician. Details of the program are available from the authors upon request.

In addition to implementing the clinical process, the PCMR provides **practice support to primary care physicians **for management of targeted patients by consolidating continuing medical education programs and developing clinical tools and documentation to ensure that practices are standardized in accordance with the guidelines. Lastly, the PCMR plans, in a perspective of integrated services networks, **to establish and consolidate links among various partners **(e.g. primary care clinics, cardiometabolic education centre, resources in specialized care, pharmacists) and especially to consolidate service pathways that facilitate primary care access to specialized health services. Moreover, mechanisms will be put in place to ensure the clinical information needed for effective patient management and monitoring circulates fluidly among professionals. The ASSSM plans to implement a computerized regional registry for chronic diseases that includes anthropometric and biochemical data on the clientele being followed. For professionals of the cardiometabolic education centres, the registry will serve as a clinical tool, while it will be used as a communication tool by attending physicians and for project evaluation.

Participating CSSS are responsible for implementation of the PCMR, and for setting up local coordination committees to oversee project planning and implementation, and to follow up on the project evaluation. In each CSSS, the local committee will set up a clinical committee responsible for the clinical tools and contents needed to implement the project. The ASSSM will provide regional leadership and organizational support required to implement the program in CSSS. Mechanisms will be put in place to encourage primary care physicians to participate (e.g. early involvement of primary care physicians in developing the program and within local coordination committees; support for the project from professional associations; meetings in primary care clinics to enlist the cooperation of physicians). Furthermore, financial compensation is available from the RAMQ (Québec's health insurance board) for general practitioners who help organize clinical projects in a CSSS territory.

### Conceptual framework for program evaluation

**Figure 1:**
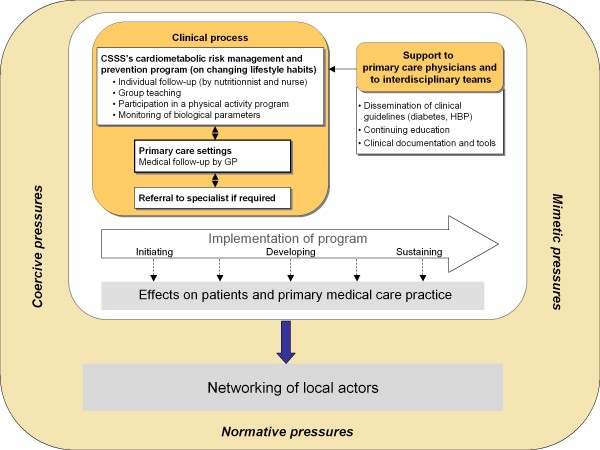
**Conceptual framework**.

The starting point for our conceptual framework (Figure 1) is the implementation of the program, which takes place over a 40-month period and includes three phases: initiation, development and sustainability. The latter takes shape by integrating PCMR activities into regular CSSS planning. The effects observed on patients and on medical practice vary depending on the degree to which the PCMR is implemented. Program implementation is influenced by a set of contextual factors that have various influences [31-33]. The ASSSM, which shares responsibility with the CSSS for meeting population needs, sponsors the project and has a determining and highly influential effect through the resources it allocates to the project. The CSSS also has a direct influence on the program due to its organizational characteristics and its structure, as well as to the role played by its human, material, technological and other resources. Moreover, by creating a critical mass of resources accessible to organizations in the territory, the CSSS introduces into the local services network incentives that can lead to greater inter-organization collaboration, which then favours the development of integrated networks. In this sense, the influence of the ASSSM and the CSSS on the development of local services networks can be seen as coercive [33,34]. Other organizations in the local network can also influence network implementation. For instance, the presence of Family Medicine Groups (FMG) can provide benchmarks and models that other medical clinics can emulate (mimetic influence). Similarly, specialized hospitals and other resources can influence the nature and degree of collaboration and networking. Finally, professional associations and their leaders can facilitate or hamper network implementation. In that respect, the Association des médecins omnipraticiens de Montréal, the Département régional de médecine générale and its local representatives as well as other leaders in the field greatly influence collaboration among local service network organizations as well as implementation of networks (normative influence). These various influences are exerted within a dynamic environment and contribute to its evolution.

### Research Questions and Objectives

#### Objective 1

To evaluate the effects of the PCMR and their persistence on patients registered in the program and the practices of their primary care physicians, by implementation site and degree of exposure to the program.

For patients, the program should result in improved lifestyle habits and better control of their diabetes and/or their hypertension. Chronic illness management and follow-up should be noticeably improved, as should patient self-management and quality of life. Closer and better coordinated follow-up should also improve certain elements of the patients' experience of care with their primary care physicians (continuity of information, comprehensiveness and perceived outcomes of care). Finally, controlling their condition is likely to lead patients to use health services more appropriately (fewer visits to emergency and fewer hospitalizations for problems related to diabetes or hypertension). In all likelihood, these effects on patients will vary depending on the setting (CSSS) and degree of exposure to the PCMR. As regards medical practice, PCMR implementation should be reflected in participation in the program (continuous medical education activities, use of clinical tools, referral of patients to cardiometabolic risk education centres), interprofessional collaboration and patient management interventions. Persistence of effects on both patients and medical practice remains to be demonstrated.

#### Objective 2

To assess the degree to which the PCMR has been implemented in each participating CSSS territory (local services network) and identify the context-related factors (coercive, normative and mimetic pressures) that could explain the level of implementation.

The aim is to answer the following research questions: To what degree has each setting implemented the program? What contextual factors facilitate or hinder program implementation? To what extent is program sustainability assured: does the PCMR fit into CSSS program activities?

#### Objective 3

To establish the relationships between the observed effects (Objective 1), degree of implementation of the program and contextual factors (Objective 2).

This objective implies the following research questions: How do variations in PCMR implementation influence the effects observed? To what extent do the contextual factors that facilitate or hinder program implementation influence the observed effects?

#### Objective 4

To assess the impact of implementing the PCMR on strengthening local services networks.

This objective concerns the following research question: To what degree does implementing this program help consolidate local networks by establishing closer and long-lasting links among service providers in the CSSS territory?

## Methods/Design

### Design

PCMR evaluation includes analysis of its implementation and of its effects. We posit that the program concept and implementation will adjust to the different CSSS settings. The proposed evaluation will use a mixed design that involves two complementary research strategies [35]. The first strategy is a quasi-experimental "before-after" design, based on a quantitative approach; it will look at the program's effects and their variations among the six settings where it is implemented. This situation corresponds to a natural experiment in which researchers have no control on the intervention being introduced.

The second strategy is consistent with a multiple case study approach, where each of the six settings constitutes a case. This strategy is based on qualitative methods and will characterize the variable represented by the implementation context. A monograph for each setting will be produced following analysis and will include data on the various indicators described below. The monographs will explain the processes that have led to the effects observed and the particular contextual conditions that can be linked to differences in implementation and effects. Adding this component to the evaluation will therefore increase both internal and external validity.

The two strategies will generate data that we will triangulate to provide a deeper explanation of the link between degree of program implementation, context and observed effects. Triangulation will be based on an interpretative analysis of the data collected and on a validation process with individuals responsible for implementing the PCMR through discussion groups that will also serve as mediums for knowledge translation and exchange.

### Indicators, data sources and analyses

#### Objective 1

To meet this objective, the indicators measuring the effects of the PCMR (Additional file 1) will be compared at different stages of the program as well as among various implementation settings. For each patient, follow-up at the cardiometabolic risk education centre determined in the PCMR will extend over 24 months.

Several of the **indicators for patients **pertain to control of patients' biological parameters (HbA1c, BP, lipid profile, body mass index, waist circumference) and lifestyle habits (diet, physical activity, smoking). These indicators include measures of change during the intervention as well as measures of attainment of clinical targets at the end of the evaluation (e.g. proportion of patients with HbA1c ≤ 7%). Data needed to calculate indicators of biological parameters and lifestyle habits for each patient will be taken from the regional computerized registry for chronic illnesses. These data will be compiled in the registry at program entry and then at the 3^rd^, 6^th^, 12^th^, 18^th ^and 24^th ^month of follow-up.

Indicators for health services utilization by patients in the PCMR, their experience of care, follow-up of diabetes and/or hypertension, self-management and quality of life will be measured using a 20-minute self-administered questionnaire, in French or English, given to patients by the nurse or nutritionist at the cardiometabolic education centre upon program entry, and at 12 and 24 months of follow-up (Additional file 2 and Additional file 3). The questionnaire will be completed on site, with support provided for patients with low literacy levels when required. A postal questionnaire will also be administered 36 months after onset of follow-up (Additional file 3). Data collected at 36 months--a year after the end of follow-up in the program--will be used to check the persistence of effects post-intervention. The first section of the questionnaire focuses on patients' utilization of health services (medical visits, hospitalizations, visits to emergency) during the previous year. The second section documents patient affiliation to the primary care physician who provides diabetes and/or hypertension follow-up and who referred him or her to the education centre, the characteristics of affiliation (duration, frequency and place of consultation), as well as certain attributes related to the patient's care experience with this physician (accessibility, continuity, comprehensiveness and perceived care outcomes). Questions in this section are adapted from the population survey questionnaire developed for a study carried out by our research team aimed at assessing the evolution of primary healthcare organizations and their performance (2005-2010) in two regions of Québec province [36]. Section 3 of the questionnaire concerns management and follow-up of chronic illnesses. These questions are adapted from the *Patient Assessment of Chronic Illness Care *questionnaire [11]. The 4^th ^section is on patient self-management and quality of life related to their diabetes and/or hypertension. Self-management is measured using questions adapted from the *Summary of Diabetes Self-Care Activities*, and assesses the frequency of self-management behaviours during a typical week (preventive behaviours--diet, physical activity, smoking abstinence--as well as compliance with medication as prescribed, glycemia and BP monitoring) [37]. Questions measuring patients' quality of life are adapted from the questionnaire *Audit of Diabetes Dependent Quality of Life *[38-40]. Data on patients' sociodemographic characteristics and health status are the subject of the last section of the questionnaire. Linking patient data to data collected from his or her physician will allow us to determine how primary care services are organized at the place where the patient is seen. A questionnaire will also be administered to patients who withdraw from the PCMR prematurely; it will include questions designed to document the reasons why a person drops out (Additional file 4).

Use of health services by patients in the PCMR will also be documented with medical administrative data (medical services, hospitalizations, emergency room visits), using the patient's health insurance number to match medical administrative data to data from the regional computerized registry on chronic illnesses and to his or her survey data.

Regression models will be built to assess the program's effects on patients, in terms of biological parameters, lifestyle habits, health services utilization, chronic illness follow-up, self-management, quality of life and care experience, based on the CSSS, degree of exposure to the program, patient characteristics, and organizational characteristics of primary care clinics where patients are seen. Particular attention will be paid to patients' sociodemographic characteristics (male/female, age, income, education) in terms of equity of access to services. Linear models will be constructed for continuous dependent variables (e.g. before-after intervention differences in HbA1c or BP). Logistic models will be used for dichotomous dependent variables (e.g. clinical targets achieved or not by the end of the intervention, dichotomized indices). Longitudinal analyses will enable us to model the evolution of dependent variables throughout the follow-up period. These analyses will be performed using multilevel models (Generalized Linear Mixed Models) [41] in which individual repeated observations will be nested within patients. We will thus be able to control for certain fixed subject characteristics as well as for time-varying covariates.

#### Patient recruitment and power calculation

We plan on recruiting about 250 patients a year in each participating CSSS, for a total of 3500 patients over 28 months. Assuming that recruitment rate is constant, we estimate that the 2000 patients recruited in the first 16 months could be the object, during the study, of at least three observations for data collected using questionnaires, and at least six observations for clinical data (Table 1). For the 1500 patients recruited during the last 12 months of recruitment, we will have two observations for data collected using questionnaires and at least four observations for clinical data. We expect that an initial sample of 3500 subjects, with an attrition rate of 20%, would ensure 2800 subjects available for analyses over two time measures. This sample size would allow detection of a difference in average between two territories that is about 0.14 in standard deviation units in longitudinal linear models, and about 7%, with a power of 80% at the 0.05 significance level in logistic models [42,43]. These calculations are conservatively based on the number of subjects measured twice; use of effective covariables or of a higher number of observations per subject should lead to an increase in the power of models.

**Table 1:** Patient recruitment and numbers of data collected

Number of patients	Questionnaires (services utilization, affiliation to primary care physician, chronic illness monitoring, self-management, quality of life)	**Registry data (biological measures and lifestyle habits)**
500 patients recruited in first 4 months	0, 12, 24 and 36 months	0, 3, 6, 12, 18 and 24 months

500 patients recruited in months 5 to 8	0, 12 and 24 months	0, 3, 6, 12, 18 and 24 months

500 patients recruited in months 9 to 12	0, 12 and 24 months	0, 3, 6, 12, 18 and 24 months

500 patients recruited in months 13 to 16	0, 12 and 24 months	0, 3, 6, 12, 18 and 24 months

500 patients recruited in months 17 to 20	0 and 12 months	0, 3, 6, 12 and 18 months

500 patients recruited in months 21 to 24	0 and 12 months	0, 3, 6 and 12 months

500 patients recruited in months 25 to 28	0 and 12 months	0, 3, 6 and 12 months

**Indicators concerning the impact on medical practice **will be measured for participating general practitioners using a 15-minute self-administered mail-in questionnaire, in French or in English, the first time one of his or her patients attends the cardiometabolic risk education centre, and then at 12, 24 and 36 months (Additional file 5 and Additional file 6). In addition to questions pertaining to physicians' sociodemographic and professional characteristics, the questionnaire also documents the organizational characteristics of their clinics using questions from the 2010 survey of primary care clinics used in our previous project [36]. One section of the questionnaire deals with physicians' practices regarding management of patients with diabetes or hypertension; these questions are adapted from the questionnaire by Nutting et al. [44]. The questionnaire for physicians administered at 12, 24 and 36 months includes several questions on their assessment of the PCMR: participation in the program, impacts on their patients and on their practice, relationships with specialized services, strengths and weaknesses of the program, as well as barriers and facilitating factors. The data analysis strategy will be similar to the one used to assess the effects of the program on patients. Physician and clinic characteristics will be utilized as covariables to construct regression models that measure the program's effects on their management of chronic diseases and their perceptions of the effects on patients and on their own practice.

#### Objective 2

Two elements are required to meet the goals. The first consists in assessing the level of PCMR implementation in various CSSS based on four criteria: 1) completeness of the components with regard to the program initially planned by the ASSSM; to assess the degree of implementation, we will measure the differences between the program planned by the ASSSM and the one actually implemented in CSSS; 2) intensity of the effort made to conduct program activities (e.g. number of training sessions, number of patients); 3) integration of the PCMR into CSSS program activities; and 4) program penetration rate among the targeted populations. This will be estimated by calculating the proportion of patients with diabetes or hypertension followed in the PCMR among patients with diabetes or hypertension in each participating CSSS territory (estimated using the medical services and hospitalizations administrative data).

The second element includes an analysis of factors influencing program implementation [45]. The goal of the analysis is to understand how and why the PCMR has evolved and adapted to its context over time. It will put into perspective what program elements that are truly implemented contribute to the effects produced and will evaluate the generalizability potential of the program to other contexts. In terms of contextual determinants of program implementation, we looked to the conceptual framework on factors to consider to produce change in organizations, developed by Champagne [46]. We consider that implementation results from the climate in which the program is implemented (itself influenced by management strategies and incentives used), the level of trust and involvement of stakeholders, the organizational structure that is likely to help or hinder implementation, and collective leadership and training. This component will also examine contextual factors that explain PCMR implementation in terms of the influences of various actors from the local services network. The ASSSM and the CSSS can have a coercive influence because of their position of authority and the significant resources they contribute. Through their professional associations, professionals collectively and individually have normative influences. In each local services network, there are also dominant organizations whose practices are considered to be exemplary by other partners, who tend to emulate them. These dominant organizations have a mimetic influence (e.g. FMG, network clinics).

Data will be collected in three phases, as per the program implementation stages. Semi-structured interviews with individuals from the ASSS and CSSS responsible for program implementation are scheduled for the beginning of the project (start-up phase). They will provide an initial portrait of regional services organization related to the activities of the program being implemented and will document the program components (objectives and purposes, roles and actors involved, resources and activities). They will also be used to define the dynamics in each implementation site. Interviews with respondents from each territory will focus on local historical contexts of interventions with people at risk of or who have diabetes or hypertension; organizational contexts of the CSSS; PCMR activities already in place and their components; local resources allocated; number of medical clinics and physicians involved; and number of patients targeted by the program. A second and third round of semi-structured interviews with local and regional respondents will be conducted 20 and 40 months after the beginning of the program. They will document PCMR activities actually implemented and degree of program implementation, activities set up to integrate the PCMR into the CSSS's regular activities, and obstacles and facilitators encountered. The questionnaires for physicians will also document barriers and facilitators. These contextual determinants will be grouped by dimension and conveyed with descriptive variables. The interview grids used at the beginning of the implementation of the project are included in Additional files 7a and 7b. Data collection tools that will be used 20 and 40 months after the beginning of the program will be constructed after analysis of the data collected at the beginning of the implementation of the project.

Based on the information collected throughout the project, a descriptive analysis of each case (implementation setting) will be performed. Six monographs, one for each setting, will describe the phases of PCMR implementation and its explanatory factors.

#### Objective 3

Two approaches will be used to attain this objective. The first is designed to complement the analysis proposed in Objective 2. Using the material compiled in the monographs, quantitative variables and categorical taxonomic variables will be created and integrated into regression analyses explaining the effects of the program. Regression models will be built to assess the influence of the variables pertaining to PCMR set up and implementation in the various CSSS on the effects observed among patients, controlling for degree of exposure to PCMR, patient characteristics and the characteristics of primary care clinics where they are seen. Given the low number of upper level units (6 CSSS), variables linked to CSSS will be considered as fixed effects in regression models. Linear regressions will be constructed for continuous dependent variables, while logistic models will be used for dichotomous dependent variables and multilevel longitudinal models for dependent variables that are the object of repeated measures. A similar analysis strategy will be used to assess the influence on medical practice of variables that reflect PCMR set up and implementation in various CSSS.

The second approach will be interpretive: from the material used to produce the monographs, participants' statements on the relationship between program effects and components will be noted and analyzed. Conclusions drawn from these two approaches will be validated in discussion groups.

#### Objective 4

This objective concerns the PCMR's influence on interorganizational collaboration and networking among local services networks. We are interested in mechanisms for collaboration that exist between CSSS and primary care organizations in the territories and health care and services providers, as well as their evolution over time. We will use the framework developed for our previous project [36]. Interorganizational collaboration within local services networks will be documented at the beginning of the project, based on the results of our previous project [36], which looked at this issue for CSSS territories in Montréal in 2010 (questionnaire in Additional file 8). A questionnaire administered to key CSSS informants 40 months after project onset will document the evolution of interorganizational collaboration within local services networks in relation to PCMR implementation (Additional file 9). By becoming a model of service integration, the PCMR is in fact likely to form a best practice that has a mimetic influence on local services network partners, and eventually lead to the creation of other networks (e.g. for COPD) and, more generally, to interorganizational collaboration. The questionnaire will be adapted from questionnaires used in our previous project [36] to explore territorial contexts. Questions on the PCMR's influence on interorganizational collaboration and networking within local services networks will also be included in the final questionnaire for physicians participating in the program.

### Ethical considerations

The project has received approval from the ASSSM's research ethics committee.

### Strengths and limitations of the proposed project

Our project has both strengths and weaknesses. Strictly speaking, for a comparative analysis, the number of CSSS in our study is relatively small. However, for a multiple case analysis strategy, this number is sufficient to show contrasts and can rather be seen as a strength of our study. The absence of a control group is a limitation but in this type of study, it is impossible to use an experimental design; we replaced it with a quasi-experimental design [47] and a hybrid analysis strategy that combines quantitative and qualitative aspects. Triangulation increases internal but also external validity, which is often lacking in experimental designs. Moreover, although the 40-month observation period is long enough to show the effects of the PCMR and to document the implementation process, it may not be sufficient to estimate, over the longer term, the sustainability of the effects and of activities undertaken.

Some of the strong points of our study include the fact that it will enable us to fill certain gaps in knowledge and, more particularly, to evaluate the application of the CCM to a primary care "case management" approach rather than to "disease management". It will also allow us to evaluate CCM implementation from a systemic perspective based on the will of the ASSSM to completely implement the proposed program in all Montréal CSSS. Mostly, our study will identify the contextual factors that can modulate the effects of the CCM and that can be associated with successful implementation and with the effectiveness of such a program. These contextual elements will enable us to extrapolate our results to other contexts with similar conditions as well as to other chronic illnesses. The study will also allow us to assess the impact of implementing a program inspired from the CCM on consolidation of local services networks. Finally, in terms of the methodology, we should add that other strengths of the project are sample size, repeated number of observations and use of numerous tools (questionnaires) that have either been validated or used in our previous studies.

### Knowledge translation and exchange plan, and partnerships

Since this project involves decision makers, clinicians and researchers, the knowledge translation and exchange plan is built into the study and consists in having mutual exchanges throughout the duration of the study. This close proximity fosters knowledge exchange and appropriation. The evaluation project is formative and many knowledge transfer activities are planned throughout the study.

To actualize some of these knowledge transfer activities, two regional steering committees (a select committee composed of regional decision-making/management/clinical representatives for which four or five meetings a year are planned, and a broader committee including local representatives that will meet twice a year) will be set up to support the research team during various stages of the project. Moreover, local coordination and clinical committees will be potential sources of information to document the degree of implementation and of venues to engage in dialogue to validate and interpret the results obtained. Halfway through the project, we will organize a meeting of local and regional decision-makers, managers and clinicians, who will be asked to react to results regarding the degree of program implementation in various CSSS and identification of factors that facilitate or hamper implementation.

We will also set up an advisory committee composed of representatives from among the project researchers and decision makers as well as other nationally and internationally known researchers and decision makers (some from other provinces) with expertise in research on primary care services organization and chronic illnesses. We plan to have two meetings with this committee to help us focus data collection and result analyses to promote the project's specific contribution to current knowledge and increase the potential to adapt our results to other contexts.

At the end of the project and with the help of the Institut national de santé publique du Québec (INSPQ), we will set up an exchange forum that will allow us to add more decision makers and researchers to the advisory committee; they will enhance adaptation of research results in the contexts of Québec and of other Canadian provinces, as well as internationally. The forum will present an opportunity to set up a more permanent structure for collaboration among researchers and decision makers, and thus enhance the outcome and value of our research by developing prolonged and sustainable partnerships. Our team has proved its capacity to implement such a structure. The exchange forum will bring together decision makers from the areas of primary care services organization and chronic diseases (from clinics, and local and provincial decision makers) as well as researchers from various backgrounds with whom the research team has collaborated for several years.

Moreover, we will benefit from the network the INSPQ developed through the Initiative sur le partage des connaissances et le développement des compétences, a consortium of provincial, regional and local partners (including CSSS) whose mandate is to implement population-based responsibility. Results will also be presented at conferences in Québec (e.g. Journées annuelles de santé publique, Rendez-vous de la gestion des maladies chroniques) as well as at national and international meetings. A research report will be written and scientific articles submitted for publication.

## Competing interests

The authors declare that they have no competing interests.

## Authors' contributions

An evaluation of the implementation of integrated services networks requires a range of research experience and skills. The research team includes knowledgeable researchers who have extensive research experience pertaining to health services organization and performance, and experience in developing innovative population-based interventions.

SP, RP, PT are members of a research team (ESPSS, a health services and population health team) linked to the Direction de santé publique (DSP) de Montréal and to the Institut national de santé publique du Québec (INSPQ). They have carried out many studies on primary care, its organization and its contribution to population health, including the following three: 1) Accessibility and Continuity of Care: A study of primary care services in Québec (Pineault et al. 2009); 2) Association of primary health care service models with perceived health status, utilisation of health services, ability for self-care and perceived quality of services in patients with chronic disease (Feldman et al., 2005-2008); and 3) L'évolution de l'organisation et de la performance des services de 1^re ^ligne (2005-2010) dans deux régions du Québec: Montréal et Montérégie (Levesque et al., 2009-2012). RP, coprincipal investigator is also Fellow in the Department of Social and Preventive Medicine at Université de Montréal. He will be mentor to the research team. SP is co-principal investigator. She has expertise in clinical preventive services, chronic illness management and surveys. PT is the nominated principal investigator and a researcher in epidemiology at McGill University. He possesses special expertise in the analysis of large medical administrative databases. The three principal investigators, affiliated with the Research Centre of the Centre Hospitalier de l'Université de Montréal (CHUM), have been lead researchers or co-researchers or have collaborated in the three projects listed above, and will be involved in all stages of the project.

The research team also includes co-researchers associated with the ESPSS team. RBDS, health economist and postdoctoral trainee with the ESPSS team, is co-investigator on project 3. Her primary interests are general practitioners' practice methods and analyses performed with medical administrative databases. MH has been coordinator for project 1 and co-investigator on projects 1 and 3, to which she has brought an expertise in primary care organization, integration of services and multidimensional analyses. She will be involved in the program implementation analysis.

All the authors have been involved in drafting the manuscript; they also read and approved the final manuscript.

## Pre-publication history

The pre-publication history for this paper can be accessed here:

http://www.biomedcentral.com/1471-2296/12/126/prepub

## Supplementary Material

Additional file 1 **List of indicators and measures**. This file lists the indicators and measures related to the effects of the program on patients and medical practice along with their sources of data. Click here for file

Additional file 2 **Questionnaire for patients at their entry into the program**. Questions relate mainly to utilization of health services, experience of care with the primary care physician, management and follow-up of chronic illnesses, self-management and quality of life related to diabetes and/or hypertension, sociodemographic characteristics and health status. Click here for file

Additional file 3 **Questionnaire for patients at 12, 24 and 36 months after entry into the program**. This questionnaire includes questions on utilization of health services, experience of care with the primary care physician, management and follow-up of chronic illnesses, self-management and quality of life related to diabetes and/or hypertension. Click here for file

Additional file 4 **Questionnaire for patients who dropped out of the program during follow-up**. Includes questions aimed at documenting the reasons for dropping out. Click here for file

Additional file 5 **Questionnaire for primary care physicians at registration of a 1^st ^patient to the cardiometabolic risk program**. This questionnaire contains questions pertaining to physicians' sociodemographic and professional characteristics, to the organizational characteristics of their clinics and to the physicians' practices regarding management of patients with diabetes or hypertension. Click here for file

Additional file 6 **Questionnaire for primary care physicians 12-24-36 months after registration of a 1^st ^patient to the cardiometabolic risk program**. This questionnaire includes questions on their assessment of the program: participation in the program, impacts on their patients and on their practice, relationships with specialized services, strengths and weaknesses of the program, as well as barriers and facilitating factors. Click here for file

Additional files 7 **Interview guide with individuals in charge of program implementation**. These files contain discussion themes for the semi-structured interviews conducted at the beginning of the project, with individuals from the ASSS and CSSS who are responsible for program implementation. Discussion themes include an initial portrait of the services organization related to the activities of the program being implemented, the program components (objectives and purposes, role and actors involved, resources and activities), the dynamics in each implementation site. Click here for file

Additional file 8 **CSSS Questionnaire**. This file contains the questionnaire used to document interorganizational collaboration within local services networks in a previous project [36]. Click here for file

Additional file 9 **CSSS Questionnaire - End of project evaluation**. This questionnaire, administered to key CSSS informants 40 months after project onset, will document the evolution of interorganizational collaboration within local services networks in relation to the program implementation. Click here for file
