# Supplementary material for: Evaluation of the implementation of an integrated primary care network for prevention and management of cardiometabolic risk in Montréal
Source: BMC Fam Pract. 2011 Nov 10;12:126. doi: 10.1186/1471-2296-12-126 (PMC3282661; doi:10.1186/1471-2296-12-126)
Supplement: Additional file 1 — List of indicators and measures. This file lists the indicators and measures related to the effects of the program on patients and medical practice along with their sources of data. [file 1471-2296-12-126-S1.DOC]

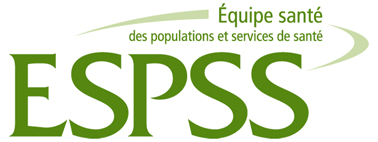
Additional file 1

**Evaluation of the implementation of an integrated primary care network for prevention and management of cardiometabolic risk in Montréal**

**List of proposed indicators related to the effects of the program**

**on patients and medical practice**

April 2011


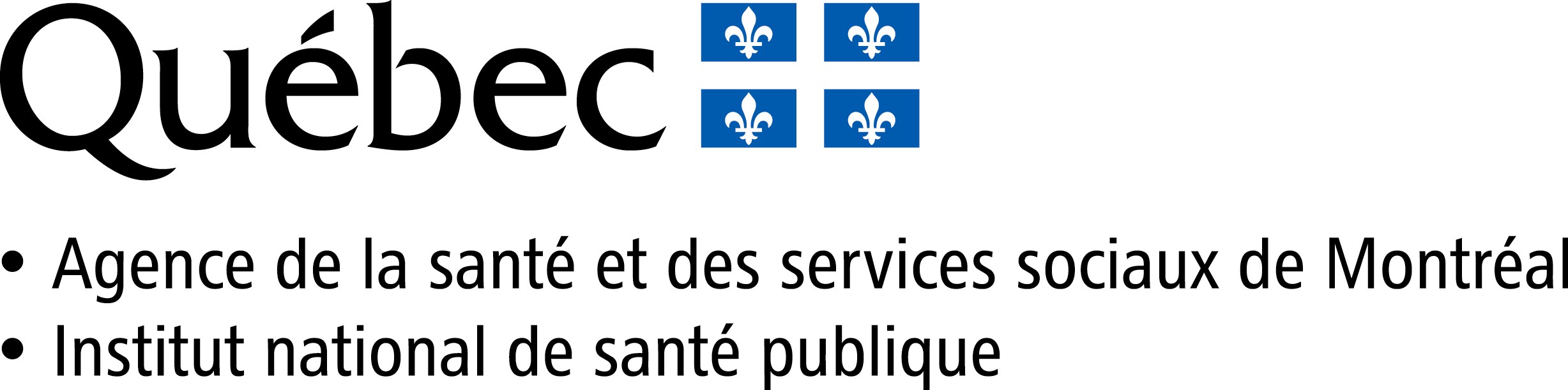


| **Dimension** | **Indicators and measures** | | **Data sources** |
| --- | --- | --- | --- |
| **Effects of the program and of its components, in terms of benefits for participating patients** | | | |
| **Clinical parameters of patients** | | Change in HbA1c | Computerized chronic disease registry  *(data to compile at patient registration in the program and then at 3, 6, 12, 18, 24 and 36 months)* |
|  |  | Attainment of clinical target of HbA1c ≤ 7% (at 12, 24, 36 months) |  |
|  |  | Change in systolic BP (mmHg) |  |
|  |  | Change in diastolic BP (mmHg) |  |
|  |  | Attainment of clinical target of BP ≤ 140/90 (≤ 130/80 for diabetics) (at 12, 24, 36 months) |  |
|  |  | Change in BMI (kg/m^2^) |  |
|  |  | Change in waist circumference |  |
|  |  | Change in lipid profile |  |
|  |  | Attainment of clinical target of LDL-C level of ≤ 2.0 mmol/L (at 12, 24, 36 months) |  |
|  |  | Attainment of clinical target of TC/HDL-C ratio < 4.0 (at 12, 24, 36 months) |  |
| **Compliance to medication** | | Compliance to hypoglycemic and/or antihypertensive medication |  |
| **Lifestyle habits** | | Change in level of physical activity (calculation based on number of days/week with at least 30 minutes of physical activity and intensity of the activity) |  |
|  |  | Attainment of clinical target for physical activity (at 12, 24, 36 months) |  |
|  |  | Change in eating habits (consumption of carbohydrates, fats, fruit and vegetables, alcohol use, and salt intake) |  |
|  |  | Attainment of clinical target for eating habits (at 12, 24, 36 months) |  |
|  |  | Change in smoking status |  |
|  |  | Attainment of clinical target of non-smoking status (at 12, 24, 36 months) |  |
| **Management of chronic disease** | | Indicators of the *Patient Assessment of Chronic Illness Care* tool (Glasgow et al., 2005) | Survey of patients followed in the program  *(data to compile at patient registration in the program and then at 12, 24 and 36 months)* |
| **Self-management of chronic disease** | | Self-management indicators adapted from the *Summary of Diabetes Self-Care Activities* tool (Toobert et al. 2000)  Weekly occurrence of   - preventive behaviours linked to metabolic risk (eating habits, physical activity, smoking abstention) - compliance to medication prescribed - monitoring of blood glucose levels and blood pressure |  |
| **Quality of life** | | Quality of life indicators adapted from *Audit of Diabetes Dependent Quality of Life* tool (Bradley et al. 1999)  Impact of diabetes or hypertension on the patient’s   - working life, social life, sexual life - physical and sport activities, vacations, recreational activities, travel - worries about the future - motivation - enjoyment of food |  |
| **Care experience** | | Patient's care experience with the attending primary care physician in the past year   - First contact accessibility - Affiliation and follow-up continuity - Informational continuity - Comprehensiveness of care - Perception of care outcomes | Survey of patients followed in the program  *(data to compile at patient registration in the program and then at 12, 24 and 36 months)* |
| **Health services utilization** | | Use of health services for a health problem linked to diabetes or hypertension in the past year   - Number of visits with general practitioners - Number of visits with specialists - Number of visits with other health professionals - Number of visits to emergency room - Number of hospital admissions | Survey of patients followed in the program  *(at patient registration in the program and then at 12, 24 and 36 months)*  Medical administrative data (hospitalizations, medical services) |
| **Level of exposure and level of conformity to the clinical process** | | Individual follow-up (number of meetings)   - Nurse - Nutritionist | Computerized chronic disease registry  *(data to compile 12 and 24 months after patient registration in the program)* |
|  |  | Group education (number of encounters) |  |
|  |  | Participation in physical activity program (number of encounters) |  |
|  |  | Adherence to the schedule proposed in the program |  |
|  |  | Use of additional services (number of visits, number of phone calls) |  |
|  |  | Use of complementary services (e.g. smoking cessation services) |  |
| **Characteristics of patients** (control variables in explanatory analyses) | | Sociodemographic and health characteristics of patients followed in the program   - Age, sex, level of education, economic status - Perception of state of health - Comorbidities | Survey of patients followed in the program  *(data to compile at patient registration in the program and then at 12, 24 and 36 months)* |
|  |  | - Duration of diabetes and/or hypertension at entry in the program | Computerized chronic disease registry  *(data to compile at patient registration in the program)* |
| **Waiting time for the program** | | - Delay between reception of the referral to the program and the 1^st^ visit |  |

| **Effects of the programs and its components, in terms of practical support for participating physicians** | | |
| --- | --- | --- |
| **Physicians' perceptions of the impact of the intervention on the state of health of patients referred to cardiometabolic services in CSSS** | Physician's perception, for most patients referred to the program, of   - disease control - motivation to control their illness - information about their illness | Survey of primary care physicians participating in the program  *(data to compile 12, 24 and 36 months after registration of a first patient in the program)* |
|  | Changes in lifestyle habits for most of the patients referred to the program |  |
|  | Changes in self-management of illness for most patients referred to the program |  |
|  | Use of health services by most patients referred to the program   - Use of emergency for conditions associated with their diabetes or hypertension - Hospitalization for conditions associated with their diabetes or hypertension |  |
| **Impact of the program on the medical practice** | Participation in the program   - Number of patients referred to the cardiometabolic risk education centre - Referral of most eligible patients with diabetes or hypertension |  |
|  | Interprofessional collaboration   - Clinical feedback about all patients referred to the education centre - Referral of patients with diabetes or hypertension to specialists - Clinical feedback from the specialists consulted |  |
|  | Perception of the usefulness of the program for medical practice regarding   - usefulness of continuing professional development activities - usefulness of clinical tools (e.g. guideline summaries, documents for patients) - usefulness of patient follow-up jointly with the education centre (complementarity with regard to physician follow-up, alleviation of the physician’s task of educating patients, better patient follow-up) |  |
|  | Improvement of knowledge regarding   - management and follow-up of patients with diabetes or hypertension - resources available for patients with diabetes or hypertension |  |
|  | Management and follow-up of patients with diabetes and hypertension (questionnaire by Nutting et al., 2007)   - Use of a registry to identify and/or track patient care - Use of a recall system for required visits or exams - Follow-up of patients by telephone between clinic visits - Use of published practice guidelines as the basis for treatment plan - Involvement of office staff in identifying and reminding patients requiring follow-up or other services - Assistance for patients in establishing self-management care objectives - Referral of patients to professional in the clinic or outside the clinic for education about their chronic illnesses - Use of flow sheets in medical files to track critical elements of care | Survey of primary care physicians participating in the program  *(data to compile at registration of a first patient in the program and 12, 24 and 36 months after)* |
| **Characteristics of participating primary care physicians** (control variables in explanatory analyses) | Organizational characteristics of the clinic   - Type of clinic (private, FMG, CLSC) - Size of the organization (number of physicians) - Information technologies - Proportion of walk-in visits - Services offered - Nurse's role, if applicable | Survey of primary care physicians participating in the program  *(data to compile at registration of a first patient in the program)* |
|  | Personal and professional characteristics of physicians   - Sex, number of years of experience - Type of practice (% in primary care clinic, % in walk-in primary care clinic, number of patients/week in primary care clinic) - Proportion of patients with diabetes or hypertension among the clientele |  |
