# Supplementary material for: Evaluation of the implementation of an integrated primary care network for prevention and management of cardiometabolic risk in Montréal
Source: BMC Fam Pract. 2011 Nov 10;12:126. doi: 10.1186/1471-2296-12-126 (PMC3282661; doi:10.1186/1471-2296-12-126)

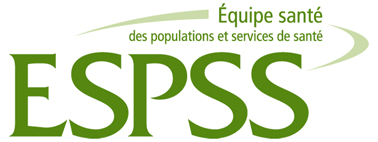
**Additional file 2**

File number: ___________ Site number: ___________

**Evaluation of the implementation of an integrated primary care network for prevention and management of cardiometabolic risk in Montréal**

**Questionnaire for patients**

**at program entry**

February 2011


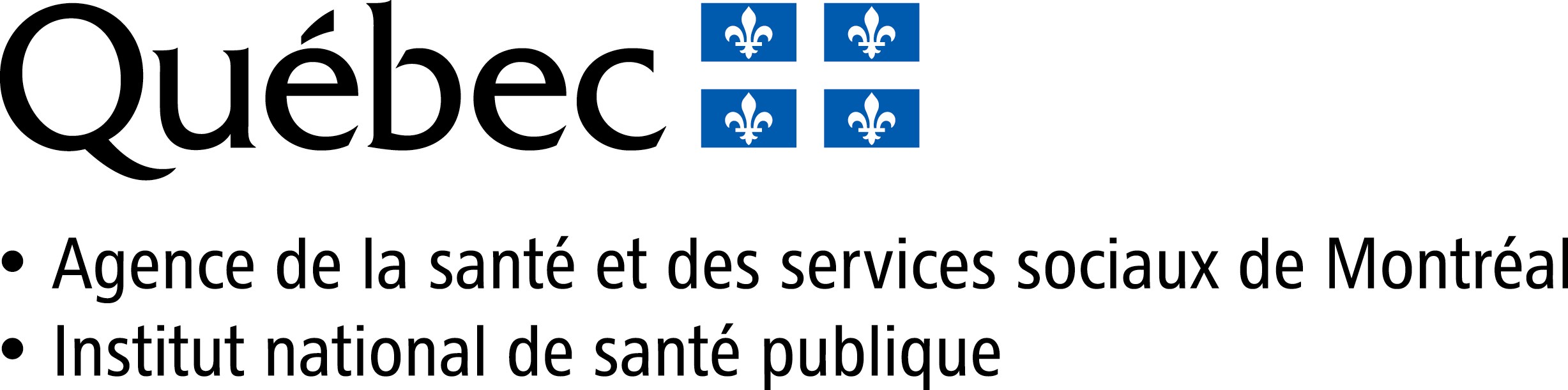


Hello,

Please read each of the following questions carefully. Give only one answer to each question. If you have any questions, ask the person who gave you the questionnaire.

Thank you for answering this questionnaire. Your participation is very important to us.

The research team

**Section A – Health Services Utilization**

A1 In the past 12 months, how many times did you see a **general practitioner (or a family doctor) for a problem related to your diabetes or high blood pressure**, other than during a hospitalization or visit to the hospital emergency room? (*If never, enter 0*)

| 1. The general practitioner that you usually see for your  diabetes or high blood pressure | Number of times by appointment: _____  Number of times without appointment: _____ |
| --- | --- |
| 1. Another general practitioner | Number of times by appointment: _____  Number of times without appointment: _____ |

A2 In the past 12 months, how many times did you see a **specialist for a problem related to your diabetes or high blood pressure**, other than during a hospitalization or visit to the hospital emergency room?

|  | Indicate the number of times: (*If never, enter 0*) |
| --- | --- |
| 1. Cardiologist |  |
| 1. Endocrinologist |  |
| 1. Ophthalmologist |  |
| 1. Nephrologist |  |
| 1. Other specialist (*indicate which one or ones:* _____________________________________) |  |

A3 In the past 12 months, how many times did you go to a **hospital emergency room** **for a problem related to your diabetes or high blood pressure?** (*If never, enter 0*) _____

A4 In the past 12 months, how many times were you **hospitalized** **for a problem related to your diabetes or high blood pressure?** (*If never, enter 0*) _____

A5 In the past 12 months (excluding today’s visit), how many times did you consult (during a visit or by phone) one of the following **health professionals** **for your diabetes or high blood pressure**?

|  | Indicate the number of times: (*If never, enter 0*) |
| --- | --- |
| 1. Nutritionist |  |
| 1. Nurse |  |
| 1. Kinesiologist (physical activity specialist) |  |
| 1. Other professional (*indicate which one or ones:* _____________________________________) |  |

A6 In the past 12 months, how many times did you participate in **group sessions** **for your diabetes or high blood pressure?**

|  | Indicate the number of times: (*If never, enter 0*) |
| --- | --- |
| 1. At the CLSC |  |
| 1. Elsewhere |  |

**Section B – Care Experience with the General Practitioner**

The following questions are about your visits to **your doctor** **(general practitioner or family doctor) who is treating you for your diabetes or high blood pressure**. The information will be kept anonymous and confidential, and will not be communicated to your doctor.

B1 How long have you been seeing this doctor?

1 Less than 2 years

2 2 to 5 years

3 6 to 9 years

4 10 years or more

8 I don't know/I don't remember

B2 Do you usually see this doctor …

1 at a clinic or doctor's office (including a Family Medicine Group or FMG)

2 at a CLSC

3 at an FMU (Family Medicine Unit) clinic

4 at a hospital emergency room

5 at a doctor's office in an independent seniors' residence

6 during home care visits

7 others (*specify:* _________________________________________________________)

8 I don't know/I don't remember

B3 Do you consider this doctor to be your family doctor?

1 Yes

2 No

8 I don't know/I don't remember

Think about **your experience over the past 12 months**. For each of the following statements, indicate whether it applies always, often, sometimes or never to **the place where you usually go to see your doctor for your diabetes or high blood pressure**.

|  |  | Always | Often | Sometimes | Never | I don't know/  I don't remember |
| --- | --- | --- | --- | --- | --- | --- |
| B4 | When you go to this place for your diabetes or high blood pressure, you see the same doctor | 1 | 2 | 3 | 4 | 8 |
| B5 | At this place, if your doctor isn’t available, you could see another doctor for your diabetes or high blood pressure | 1 | 2 | 3 | 4 | 8 |
| B6 | If you had to see a doctor for a new health problem, you would go to this place first | 1 | 2 | 3 | 4 | 8 |

B7 When you need to see your doctor for a problem related to your diabetes or high blood pressure, in general, how long does it take to see the doctor by appointment?

1 Less than 2 weeks

2 2 to 4 weeks

3 1 to 3 months

4 4 months or more

5 I don't know because my appointments are always pre-scheduled

6 I don't know because I never make an appointment

8 I don't know/I don't remember

B8 If you needed immediate or emergency care, how long would it take to see a doctor at this place?

1 Less than 24 hours

2 1 to 2 days

3 3 to 4 days

4 5 days or more

8 I don't know/I don't remember

We would now like to know **your opinion about how accessible is the place where you usually see your doctor**. For each of the following statements, indicate if you strongly agree, somewhat agree, agree a little or don't agree at all.

|  |  | Strongly agree | Somewhat | A little | Do not agree at all | I don't know/ I don't remember |
| --- | --- | --- | --- | --- | --- | --- |
| B9 | Opening hours are convenient | 1 | 2 | 3 | 4 | 8 |
| B10 | It's easy to reach someone at this place by telephone to make an appointment | 1 | 2 | 3 | 4 | 8 |
| B11 | It's easy to get an appointment at this place | 1 | 2 | 3 | 4 | 8 |
| B12 | It's easy to talk to a doctor or nurse by telephone at this place | 1 | 2 | 3 | 4 | 8 |

Still keeping in mind **your experience over the past 12 months** **at the place where you usually see your doctor for your diabetes or high blood pressure**, indicate if you strongly agree, somewhat agree, agree a little or don't agree at all.

| At this place … | | Strongly agree | Somewhat | A little | Do not agree at all | I don't know/ I don't remember |
| --- | --- | --- | --- | --- | --- | --- |
| B13 | your medical history is known (your past medical history) | 1 | 2 | 3 | 4 | 8 |
| B14 | they are aware of all the prescription drugs you take | 1 | 2 | 3 | 4 | 8 |

| The services you get at this place help you … | | Strongly agree | Somewhat | A little | Do not agree at all | I don't know/ I don't remember |
| --- | --- | --- | --- | --- | --- | --- |
| B15 | better understand your diabetes or high blood pressure | 1 | 2 | 3 | 4 | 8 |
| B16 | prevent certain health problems related to your diabetes or high blood pressure before they appear | 1 | 2 | 3 | 4 | 8 |
| B17 | better control your diabetes or high blood pressure | 1 | 2 | 3 | 4 | 8 |

| The professionals you see at this place … | | Strongly agree | Somewhat | A little | Do not agree at all | I don't know/ I don't remember |
| --- | --- | --- | --- | --- | --- | --- |
| B18 | encourage you to follow the treatments prescribed for your diabetes or high blood pressure | 1 | 2 | 3 | 4 | 8 |
| B19 | help motivate you to adopt good lifestyle habits such as quitting smoking, doing physical activity or eating better | 1 | 2 | 3 | 4 | 8 |

**The next questions are about other services you might have received.**

B20 In the **past 12 months**, did you have **lab tests (blood or urine tests) prescribed by your doctor for your diabetes or high blood pressure**?

1 Yes

2 No ⮞ *Go to B23*

8 I don't know/I don't remember ⮞ *Go to B23*

Regarding these tests, indicate whether the following statements apply always, often, sometimes or never.

|  | | Always | Often | Sometimes | Never | I don't know/  I don't remember |
| --- | --- | --- | --- | --- | --- | --- |
| B21 | Your doctor had your test or exam results the next time you saw him or her | 1 | 2 | 3 | 4 | 8 |
| B22 | Your doctor gave you explanations about the results of your tests or exams | 1 | 2 | 3 | 4 | 8 |

B23 In the **past 12 months**, did you see one or more **specialists to whom you were referred by your doctor for a problem related to your diabetes or high blood pressure**?

1 Yes

2 No ⮞ *Go to B26*

8 I don't know/I don't remember ⮞ *Go to B26*

Regarding your visits to this or these specialists indicate if …

|  | | Always | Often | Sometimes | Never | I don't know/  I don't remember | I have not seen the doctor again yet |
| --- | --- | --- | --- | --- | --- | --- | --- |
| B24 | Your doctor had the results of your visit to the specialist(s) the next time you saw him or her | 1 | 2 | 3 | 4 | 8 | 10 |
| B25 | Your doctor discussed your visit to the specialist(s) with you | 1 | 2 | 3 | 4 | 8 | 10 |

B26 In the **past 12 months**, did you see one or more **health professionals** other than physicians (e.g. nutritionist, nurse, kinesiotherapist) **to whom you were referred by your doctor for a problem related to your diabetes or high blood pressure**?

1 Yes

2 No ⮞ *Go to B29*

8 I don't know/I don't remember ⮞ *Go to B29*

Regarding your visits to this or these professionals, indicate if …

|  | | Always | Often | Sometimes | Never | I don't know/  I don't remember | I have not seen the doctor again yet |
| --- | --- | --- | --- | --- | --- | --- | --- |
| B27 | Your doctor had the results of these visits the next time you saw him or her | 1 | 2 | 3 | 4 | 8 | 10 |
| B28 | Your doctor discussed your visits to these professionals with you | 1 | 2 | 3 | 4 | 8 | 10 |

**Unmet needs for health services**

B29 In the **past 12 months**, did you feel you **needed to see your doctor for a problem related to your diabetes or high blood pressure but were unable to do so**?

1 Yes

2 No ⮞ *Go to C1*

8 I don't know/I don't remember ⮞ *Go to C1*

We would like to know why you didn't see your doctor. For each of the following statements, indicate whether or not it applies to your situation.

|  |  | Yes | No | I don't know/  I don't remember |
| --- | --- | --- | --- | --- |
| B30 | You have an appointment but you haven't seen the doctor yet | 1  *(If yes* ⮞ *go to C1)* | 2 | 8 |
| B31 | You couldn't get an appointment | 1 | 2 | 8 |
| B32 | You couldn't get around enough to actually go see a doctor | 1 | 2 | 8 |
| B33 | Your usual doctor wasn't available at the time you needed him or her | 1 | 2 | 8 |
| B34 | The waiting time before seeing your doctor was too long | 1 | 2 | 8 |
| B35 | The office hours during which your doctor was there did not suit you | 1 | 2 | 8 |
| B36 | Your health status has deteriorated too much for you to go see a doctor | 1 | 2 | 8 |
| B37 | Your problem resolved by itself | 1 | 2 | 8 |
| B38 | Other reason *Specify:_________________________________________________________________* | | | |

**Section C – Care Provision for Diabetes or High Blood Pressure**

Staying healthy can be difficult when you have a chronic illness. We would like to know about the type of help you get for your diabetes or high blood pressure **at the place you usually see** **your doctor**. Your answers will be kept confidential.

In the past 12 months, when you received care for diabetes or high blood pressure …

|  | | Almost always | Most of the time | Sometimes | Generally not | Almost never |
| --- | --- | --- | --- | --- | --- | --- |
| C1 | you were asked for your ideas when making a treatment plan | 1 | 2 | 3 | 4 | 5 |
| C2 | you were given treatment choices to think about | 1 | 2 | 3 | 4 | 5 |
| C3 | you were asked to talk about problems with your medications or their effects | 1 | 2 | 3 | 4 | 5 |
| C4 | you were given a written list of things you should do to improve your health | 1 | 2 | 3 | 4 | 5 |
| C5 | you found that the care you received was well organized | 1 | 2 | 3 | 4 | 5 |
| C6 | you were shown how what you do to take care of your illness affects your health condition | 1 | 2 | 3 | 4 | 5 |
| C7 | you were asked to talk about your goals in caring for your illness | 1 | 2 | 3 | 4 | 5 |
| C8 | you were given help to set specific goals to improve your diet or physical activity | 1 | 2 | 3 | 4 | 5 |
| C9 | you were given a copy of your treatment plan | 1 | 2 | 3 | 4 | 5 |
| C10 | you were encouraged to go to a specific group or class to help you cope with your chronic illness | 1 | 2 | 3 | 4 | 5 |

In the past 12 months, when you received care for diabetes or high blood pressure …

|  | | Almost always | Most of the time | Sometimes | Generally not | Almost never |
| --- | --- | --- | --- | --- | --- | --- |
| C11 | you were asked questions, either directly or through a questionnaire, about your lifestyle habits | 1 | 2 | 3 | 4 | 5 |
| C12 | your values and traditions were considered when treatments were recommended | 1 | 2 | 3 | 4 | 5 |
| C13 | you were given help to make a treatment plan that you could apply in your daily life | 1 | 2 | 3 | 4 | 5 |
| C14 | you were helped to prepare to deal with your illness even in hard times | 1 | 2 | 3 | 4 | 5 |
| C15 | you were asked how your chronic illness affects your life | 1 | 2 | 3 | 4 | 5 |
| C16 | you were contacted after a visit to see how things were going | 1 | 2 | 3 | 4 | 5 |
| C17 | you were encouraged to attend programs in the community that could help you | 1 | 2 | 3 | 4 | 5 |
| C18 | you were referred to a dietician or health educator | 1 | 2 | 3 | 4 | 5 |
| C19 | you were told how your visits with other types of doctors (e.g. specialist) help your treatment | 1 | 2 | 3 | 4 | 5 |
| C20 | you were asked how your visits with other doctors were going | 1 | 2 | 3 | 4 | 5 |

**Section D – Self-Care and Quality of Life**

The following questions are about things you did to better control your diabetes or high blood pressure during the past 7 days. If you were sick during the past 7 days, please think back to the last 7 days when you weren't sick.

| *For each statement, indicate the number of days:* | | 0 | 1 | 2 | 3 | 4 | 5 | 6 | 7 |
| --- | --- | --- | --- | --- | --- | --- | --- | --- | --- |
| D1 | In the past 7 days, indicate how many days a week you ate healthy food | 0 | 1 | 2 | 3 | 4 | 5 | 6 | 7 |
| D2 | In the past 7 days, indicate how many days a week you did at least 30 minutes of physical activity (including walking) | 0 | 1 | 2 | 3 | 4 | 5 | 6 | 7 |
| D3 | In the past 7 days, indicate how many days a week you smoked a cigarette (even one puff) | 0 | 1 | 2 | 3 | 4 | 5 | 6 | 7 |
| D4 | *For people with diabetes*  In the past 7 days, indicate how many days a week you tested your blood sugar as recommended | 0 | 1 | 2 | 3 | 4 | 5 | 6 | 7 |
| D5 | *For people with diabetes*  In the past 7 days, indicate how many days a week you took your diabetes medication as recommended | 0 | 1 | 2 | 3 | 4 | 5 | 6 | 7 |
| D6 | *For people with high blood pressure*  In the past 7 days, indicate how many days a week you took your blood pressure as recommended | 0 | 1 | 2 | 3 | 4 | 5 | 6 | 7 |
| D7 | *For people with high blood pressure*  In the past 7 days, indicate how many days a week you took your high blood pressure medication as recommended | 0 | 1 | 2 | 3 | 4 | 5 | 6 | 7 |

Indicate whether you strongly agree, somewhat agree, agree a little or don't agree at all with the following statements.

|  |  | Strongly agree | Somewhat | A little | Do not agree at all | I don't know/ I don't remember |
| --- | --- | --- | --- | --- | --- | --- |
| D8 | I know a fair bit about my diabetes or high blood pressure | 1 | 2 | 3 | 4 | 8 |
| D9 | I know a fair bit about my diabetes or high blood pressure treatment (including medications) | 1 | 2 | 3 | 4 | 8 |
| D10 | I’m able to detect signs or symptoms indicating a change in the evolution of my diabetes or high blood pressure (e.g. blood test sugar, blood pressure, …) | 1 | 2 | 3 | 4 | 8 |
| D11 | I know what to do if those signs or symptoms appear (e.g. modify my medication, change my diet, contact a health professional, …) | 1 | 2 | 3 | 4 | 8 |

| If you didn't have diabetes or high blood pressure... | | a lot better | quite a bit better | slightly better | the same |
| --- | --- | --- | --- | --- | --- |
| D12 | your quality of life would be... | 1 | 2 | 3 | 4 |
| D13 | your employment or career opportunities would be... | 1 | 2 | 3 | 4 |
| D14 | your social life (family relationships, friendships) would be... | 1 | 2 | 3 | 4 |
| D15 | your sex life would be... | 1 | 2 | 3 | 4 |
| D16 | your sporting, holiday, travel or leisure opportunities would be... | 1 | 2 | 3 | 4 |
| D17 | long-term plans for you, your family or close friends (e.g. health, independence, income) would be... | 1 | 2 | 3 | 4 |
| D18 | your motivation to achieve things would be... | 1 | 2 | 3 | 4 |
| D19 | your capacity to do things physically would be... | 1 | 2 | 3 | 4 |
| D20 | your enjoyment of food would be... | 1 | 2 | 3 | 4 |

Indicate, in the following table, how important these various aspects are to you.

|  |  | Very important | Important | Quite important | Not at all important |
| --- | --- | --- | --- | --- | --- |
| D21 | your employment or career opportunities | 1 | 2 | 3 | 4 |
| D22 | your social life (family relationships, friendships) | 1 | 2 | 3 | 4 |
| D23 | your sex life | 1 | 2 | 3 | 4 |
| D24 | your sporting, holiday, travel or leisure opportunities | 1 | 2 | 3 | 4 |
| D25 | long-term plans for you, your family or close friends (e.g. health, independence, income) | 1 | 2 | 3 | 4 |
| D26 | your motivation to achieve things | 1 | 2 | 3 | 4 |
| D27 | your capacity to do things physically | 1 | 2 | 3 | 4 |
| D28 | your enjoyment of food | 1 | 2 | 3 | 4 |

**Section E – Health Status**

There are just a few more questions about your health status.

E1 In general, would you say that your health is...

1 excellent

2 very good

3 good

4 average

5 poor

E2 Has a doctor ever told you that you have a heart disease or heart problem (e.g. angina, infarct, arrhythmia, prior heart operation, heart failure…)?

1 Yes

2 No

8 I don't know/I don't remember

E3 Has a doctor ever told you that you have asthma?

1 Yes

2 No

8 I don't know/I don't remember

E4 Has a doctor ever told you that you have chronic bronchitis, emphysema or chronic obstructive pulmonary disease?

1 Yes

2 No

8 I don't know/I don't remember

E5 Has a doctor ever told you that you have rheumatism, arthritis or osteoarthritis?

1 Yes

2 No

8 I don't know/I don't remember

E6 Have you ever had a cerebral vascular accident such as thrombosis or stroke?

1 Yes

2 No

8 I don't know/I don't remember

E7 In the past 12 months, did you see a doctor for a mental health problem (for instance, depression or anxiety)?

1 Yes

2 No ⮞ *Go to E9*

8 I don't know/I don't remember ⮞ *Go to E9*

E8 Can you tell us what the exact problem was? *(If more than one, indicate only the main problem)*

1 Depression

2 Burn-out

3 Bipolar or manic-depressive disorder

4 Anxiety or phobia

5 Another problem *Specify:_______________________________________________________*

8 I don't know/I don't remember

E9 Are you currently being followed for cancer?

1 Yes

2 No

8 I don't know/I don't remember

E10 Aside from the health problems you have mentioned, do you have other health problems for which you are being followed or treated regularly?

1 Yes

2 No ⮞ *Go to F1*

8 I don't know/I don't remember ⮞ *Go to F1*

E11 Could you indicate what the problem(s) is (are)?

**Section F – Sociodemographic Characteristics**

The last questions will be used to classify your answers.

F1 Are you …

1 a man  2 a woman

F2 How old are you?

F3 Were you born…

1 in Canada

2 outside Canada ⇨ How many years have you been living in Canada? __________

F4 What language do you speak most often at home?

1 French  2 English  3 Other

F5 What is the highest level of education you have completed or diploma you have obtained?

1 No diploma (elementary school)

2 High-school diploma

3 Diploma or certificate from a trade school or vocational school

4 Diploma from a business college

5 CEGEP diploma (or classical education)

6 Bachelor's degree

7 Master's or doctoral degree

97 Other *Specify: ________________________________________________________*

F6 Which statement best describes your main occupation over the past 6 months? Were you…

1 working full time  6 temporarily off work

2 working part time  7 receiving employment insurance (unemployment)

3 at school  8 receiving social assistance recipient (or social solidarity recipient)

4 retired  97 other *Specify: _________________________________*

5 at home

F7 For statistical purposes, is your household annual income before taxes…
*(A household includes a person or group of people from a family who live under the same roof. Roommates are not included).*

1 Less than $15,000  5 $55,000 to $75,000

2 $15,000 to $25,000  6 $75,000 to $100,000

3 $25,000 to $35,000  7 $100,000 or more

4 $35,000 to $55,000

F8 Including yourself, how many people 18 years old or older usually live in your household? *(Household excludes roommates)* ___________

F9 How many people under 18 years old usually live in your household? ___________

**Thank you for taking the time to fill out this questionnaire!**


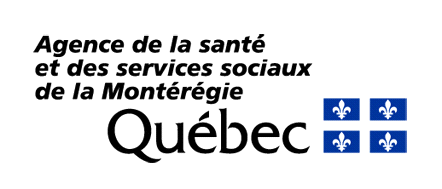

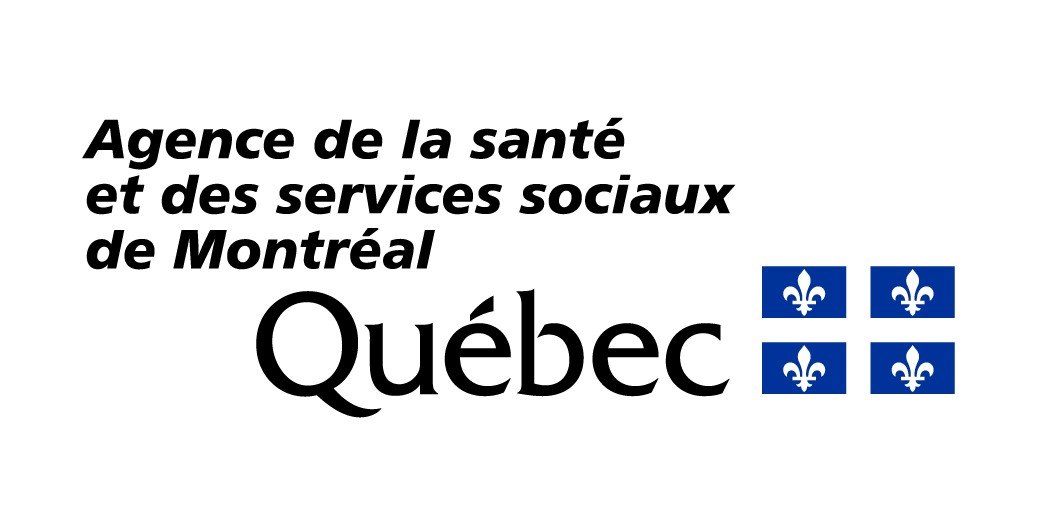


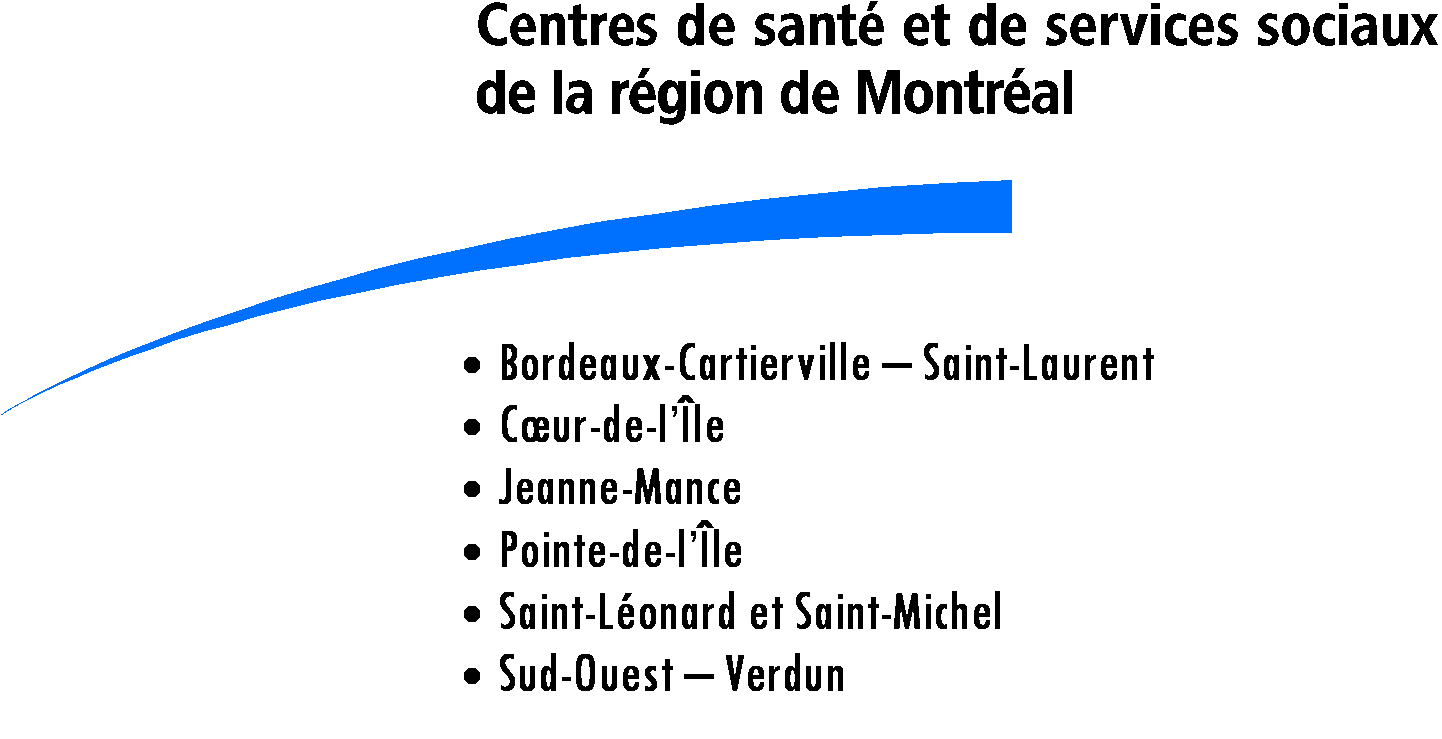

Supplement: Additional file 2 — Questionnaire for patients at their entry into the program. Questions relate mainly to utilization of health services, experience of care with the primary care physician, management and follow-up of chronic illnesses, self-management and quality of life related to diabetes and/or hypertension, sociodemographic characteristics and health status. [file 1471-2296-12-126-S2.DOC]
