# Supplementary material for: Evaluation of the implementation of an integrated primary care network for prevention and management of cardiometabolic risk in Montréal
Source: BMC Fam Pract. 2011 Nov 10;12:126. doi: 10.1186/1471-2296-12-126 (PMC3282661; doi:10.1186/1471-2296-12-126)

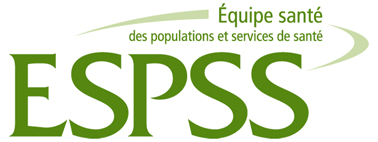
 **Additional file 3**

File number: ______________

Site number: ______________

**Evaluation of the implementation of an integrated primary care network for prevention and management of cardiometabolic risk in Montréal**

**Questionnaire for patients**

**12, 24 and 36 months after entry into the program**

April 2011


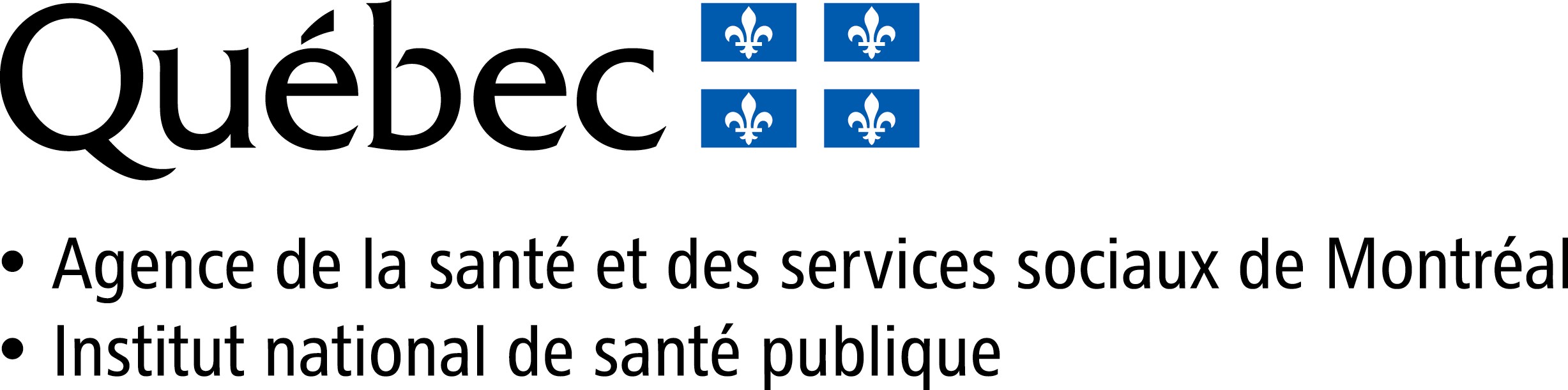


Hello,

Please, read each of the following questions carefully. Give only one answer to each question. If you have any questions, ask the person who gave you the questionnaire.

Thank you for answering this questionnaire. Your participation is very important to us.

The research team

**Section A – Health Services Utilization**

A1 In the past 12 months, how many times did you see a **general practitioner (or a family doctor) for a problem related to your diabetes or high blood pressure**, other than during a hospitalization or visit to the hospital emergency room? (*If never, enter 0*)

| 1. The general practitioner that you usually see for your  diabetes or high blood pressure | Number of times by appointment: _____  Number of times without appointment: _____ |
| --- | --- |
| 1. Another general practitioner | Number of times by appointment: _____  Number of times without appointment: _____ |

A2 In the past 12 months, how many times did you see a **specialist for a problem related to your diabetes or high blood pressure**, other than during a hospitalization or visit to the hospital emergency room?

|  | Indicate the number of times: (*If never, enter 0*) |
| --- | --- |
| 1. Cardiologist |  |
| 1. Endocrinologist |  |
| 1. Ophthalmologist |  |
| 1. Nephrologist |  |
| 1. Other specialist (*indicate which one or ones:* _____________________________________) |  |

A3 In the past 12 months, how many times did you go to a **hospital emergency room** **for a problem related to your diabetes or high blood pressure?** (*If never, enter 0*) _____

A4 In the past 12 months, how many times were you **hospitalized** **for a problem related to your diabetes or high blood pressure?** (*If never, enter 0*) _____

A5 In the past 12 months (excluding today’s visit), how many times did you consult (during a visit or by phone) one of the following **health professionals** **for your diabetes or high blood pressure**?

|  | Indicate the number of times: (*If never, enter 0*) |
| --- | --- |
| 1. Nutritionist |  |
| 1. Nurse |  |
| 1. Kinesiologist (physical activity specialist) |  |
| 1. Other professional (*indicate which one or ones:*  _____________________________________ _____________________________________) |  |

A6 In the past 12 months, how many times did you participate in group sessions at the CLSC cardiometabolic risk program (on changing lifestyle habits)? _____

**Section B – Care Experience with the General Practitioner**

Think about **your experience over the past 12 months**. For each of the following statements, indicate whether it applies always, often, sometimes or never to **the place where you usually go to see your doctor (general practitioner or family doctor) who is treating you for your diabetes or high blood pressure**. The information will be kept anonymous and confidential, and will not be communicated to your doctor.

| The services you get at this place help you … | | Strongly agree | Somewhat | A little | Do not agree at all | I don't know/ I don't remember |
| --- | --- | --- | --- | --- | --- | --- |
| B1 | better understand your diabetes or high blood pressure | 1 | 2 | 3 | 4 | 8 |
| B2 | prevent certain health problems related to your diabetes or high blood pressure before they appear | 1 | 2 | 3 | 4 | 8 |
| B3 | better control your diabetes or high blood pressure | 1 | 2 | 3 | 4 | 8 |

| The professionals you see at this place … | | Strongly agree | Somewhat | A little | Do not agree at all | I don't know/ I don't remember |
| --- | --- | --- | --- | --- | --- | --- |
| B4 | encourage you to follow the treatments prescribed for your diabetes or high blood pressure | 1 | 2 | 3 | 4 | 8 |
| B5 | help motivate you to adopt good lifestyle habits such as quitting smoking, doing physical activity or eating better | 1 | 2 | 3 | 4 | 8 |

**The next questions are about other services you might have received.**

In the **past 12 months**, you have had lab tests (blood or urine tests) done for your diabetes or high blood pressure as part of the follow-up for the CLSC program on changing lifestyle habits.

Regarding these tests, indicate whether the following statements apply always, often, sometimes or never.

|  | | Always | Often | Sometimes | Never | I don't know/  I don't remember |
| --- | --- | --- | --- | --- | --- | --- |
| B6 | Your doctor had your test or exam results the next time you saw him or her | 1 | 2 | 3 | 4 | 8 |
| B7 | Your doctor gave you explanations about the results of your tests or exams | 1 | 2 | 3 | 4 | 8 |

B8 In the **past 12 months**, did you see one or more specialists for a problem related to your diabetes or high blood pressure and to whom you were referred by your doctor or by a professional at the CLSC program on changing lifestyle habits?

1 Yes

2 No ⮞ *Go to B11*

8 I don't know/I don't remember ⮞ *Go to B11*

Regarding your visits to this or these specialists indicate if …

|  | | Always | Often | Sometimes | Never | I don't know/  I don't remember | I have not seen the doctor again yet |
| --- | --- | --- | --- | --- | --- | --- | --- |
| B9 | Your doctor had the results of your visit to the specialist(s) the next time you saw him or her | 1 | 2 | 3 | 4 | 8 | 10 |
| B10 | Your doctor discussed with you the results of your visit to the specialist(s) | 1 | 2 | 3 | 4 | 8 | 10 |

In the **past 12 months**, you saw, for your diabetes or high blood pressure, one or more health professionals at the CLSC program on changing lifestyle habits (e.g. nutritionist, nurse, kinesiotherapist).

Regarding your visits to this or these professionals, indicate if …

|  | | Always | Often | Sometimes | Never | I don't know/  I don't remember | I have not seen the doctor again yet |
| --- | --- | --- | --- | --- | --- | --- | --- |
| B11 | Your doctor had the results of these visits the next time you saw him or her | 1 | 2 | 3 | 4 | 8 | 10 |
| B12 | Your doctor discussed with you the results of your visits to these professionals | 1 | 2 | 3 | 4 | 8 | 10 |

**Section C – Care Provision for Diabetes or High Blood Pressure**

Staying healthy can be difficult when you have a chronic illness. We would like to know about the type of help you get for your diabetes or high blood pressure (including at the place you usually see your doctor and in the CLSC program on changing lifestyle habits). Your answers will be kept confidential.

In the past 12 months, when you received care for diabetes or high blood pressure …

|  | | Almost always | Most of the time | Sometimes | Generally not | Almost never |
| --- | --- | --- | --- | --- | --- | --- |
| C1 | you were asked for your ideas when making a treatment plan | 1 | 2 | 3 | 4 | 5 |
| C2 | you were given treatment choices to think about | 1 | 2 | 3 | 4 | 5 |
| C3 | you were asked to talk about problems with your medications or their effects | 1 | 2 | 3 | 4 | 5 |
| C4 | you were given a written list of things you should do to improve your health | 1 | 2 | 3 | 4 | 5 |
| C5 | you found that the care you received was well organized | 1 | 2 | 3 | 4 | 5 |
| C6 | you were shown how what you do to take care of your illness affects your health condition | 1 | 2 | 3 | 4 | 5 |
| C7 | you were asked to talk about your goals in caring for your illness | 1 | 2 | 3 | 4 | 5 |
| C8 | you were given help to set specific goals to improve your diet or physical activity | 1 | 2 | 3 | 4 | 5 |
| C9 | you were given a copy of your treatment plan | 1 | 2 | 3 | 4 | 5 |
| C10 | you were encouraged to go to a specific group or class to help you cope with your chronic illness | 1 | 2 | 3 | 4 | 5 |
| C11 | you were asked questions, either directly or through a questionnaire, about your lifestyle habits | 1 | 2 | 3 | 4 | 5 |
| C12 | your values and traditions were considered when treatments were recommended | 1 | 2 | 3 | 4 | 5 |
| C13 | you were given help to make a treatment plan that you apply in your daily life | 1 | 2 | 3 | 4 | 5 |
| C14 | you were helped to prepare to deal with your illness even in hard times | 1 | 2 | 3 | 4 | 5 |
| C15 | you were asked how your chronic illness affects your life | 1 | 2 | 3 | 4 | 5 |
| C16 | you were contacted after a visit to see how things were going | 1 | 2 | 3 | 4 | 5 |
| C17 | you were encouraged to attend programs in the community that could help you | 1 | 2 | 3 | 4 | 5 |
| C19 | you were told how your visits with other types of doctors (e.g. specialist) help your treatment | 1 | 2 | 3 | 4 | 5 |
| C20 | you were asked how your visits with other doctors were going | 1 | 2 | 3 | 4 | 5 |

**Section D – Self-Care and Quality of Life**

The following questions are about things you did to better control your diabetes or high blood pressure during the past 7 days. If you were sick during the past 7 days, please think back to the last 7 days when you weren't sick.

| *For each statement, indicate the number of days:* | | 0 | 1 | 2 | 3 | 4 | 5 | 6 | 7 |
| --- | --- | --- | --- | --- | --- | --- | --- | --- | --- |
| D1 | In the past 7 days, indicate how many days a week you ate healthy food | 0 | 1 | 2 | 3 | 4 | 5 | 6 | 7 |
| D2 | In the past 7 days, indicate how many days a week you did at least 30 minutes of physical activity (including walking) | 0 | 1 | 2 | 3 | 4 | 5 | 6 | 7 |
| D3 | In the past 7 days, indicate how many days a week you smoked a cigarette (even one puff) | 0 | 1 | 2 | 3 | 4 | 5 | 6 | 7 |
| D4 | *For people with diabetes*  In the past 7 days, indicate how many days a week you tested your blood sugar as recommended | 0 | 1 | 2 | 3 | 4 | 5 | 6 | 7 |
| D5 | *For people with diabetes*  In the past 7 days, indicate how many days a week you took your diabetes medication as recommended | 0 | 1 | 2 | 3 | 4 | 5 | 6 | 7 |
| D6 | *For people with high blood pressure*  In the past 7 days, indicate how many days a week you took your blood pressure as recommended | 0 | 1 | 2 | 3 | 4 | 5 | 6 | 7 |
| D7 | *For people with high blood pressure*  In the past 7 days, indicate how many days a week you took your high blood pressure medication as recommended | 0 | 1 | 2 | 3 | 4 | 5 | 6 | 7 |

Indicate whether you strongly agree, somewhat agree, agree a little or don't agree at all with the following statements.

|  |  | Strongly agree | Somewhat | A little | Do not agree at all | I don't know/ I don't remember |
| --- | --- | --- | --- | --- | --- | --- |
| D8 | I know a fair bit about my diabetes or high blood pressure | 1 | 2 | 3 | 4 | 8 |
| D9 | I know a fair bit about my diabetes or high blood pressure treatment (including medications) | 1 | 2 | 3 | 4 | 8 |
| D10 | I’m able to detect signs or symptoms indicating a change in the evolution of my diabetes or high blood pressure (e.g. blood test sugar, blood pressure, …) | 1 | 2 | 3 | 4 | 8 |
| D11 | I know what to do if those signs or symptoms appear (e.g. modify my medication, change my diet, contact a health professional, …) | 1 | 2 | 3 | 4 | 8 |

| If you didn't have diabetes or high blood pressure... | | a lot better | quite a bit better | slightly better | the same |
| --- | --- | --- | --- | --- | --- |
| D12 | your quality of life would be... | 1 | 2 | 3 | 4 |
| D13 | your employment or career opportunities would be... | 1 | 2 | 3 | 4 |
| D14 | your social life (family relationships, friendships) would be... | 1 | 2 | 3 | 4 |
| D15 | your sex life would be... | 1 | 2 | 3 | 4 |
| D16 | your sporting, holiday, travel or leisure opportunities would be... | 1 | 2 | 3 | 4 |
| D17 | long-term plans for you, your family or close friends (e.g. health, independence, income) would be... | 1 | 2 | 3 | 4 |
| D18 | your motivation to achieve things would be... | 1 | 2 | 3 | 4 |
| D19 | your capacity to do things physically would be... | 1 | 2 | 3 | 4 |
| D20 | your enjoyment of food would be... | 1 | 2 | 3 | 4 |

Indicate, in the following table, how important these various aspects of life are to you.

|  |  | Very  important | Important | Quite  important | Not at all important |
| --- | --- | --- | --- | --- | --- |
| D21 | your employment or career opportunities | 1 | 2 | 3 | 4 |
| D22 | your social life (family relationships, friendships) | 1 | 2 | 3 | 4 |
| D23 | your sex life | 1 | 2 | 3 | 4 |
| D24 | your sporting, holiday, travel or leisure opportunities | 1 | 2 | 3 | 4 |
| D25 | long-term plans for you, your family or close friends (e.g. health, independence, income) | 1 | 2 | 3 | 4 |
| D26 | your motivation to achieve things | 1 | 2 | 3 | 4 |
| D27 | your capacity to do things physically | 1 | 2 | 3 | 4 |
| D28 | your enjoyment of food | 1 | 2 | 3 | 4 |

**Section E – Health Status**

There are just a few more questions left about your health status.

E1 Compared with last year, would you say that your health is...

1 much better now

2 a bit better now

3 about the same

4 not quite as good now

5 much worse now

E2 In the past 12 months, has a doctor ever told you that you have a heart disease or heart problem (e.g. angina, infarct, arrhythmia, prior heart operation, heart failure…)?

1 Yes

2 No

8 I don't know/I don't remember

E3 In the past 12 months, have you had a cerebral vascular accident such as thrombosis or stroke?

1 Yes

2 No

8 I don't know/I don't remember

E4 In the past 12 months, did you see a doctor for a mental health problem (for instance, depression or anxiety)?

1 Yes

2 No ⮞ *Go to E6*

8 I don't know/I don't remember ⮞ *Go to E6*

E5 Can you tell us what the exact problem was? *(If more than one, indicate only the main problem)*

1 Depression

2 Burn-out

3 Bipolar or manic-depressive disorder

4 Anxiety or phobia

5 Another problem *Specify:_______________________________________________________*

8 I don't know/I don't remember

E6 Are you currently being followed for cancer?

1 Yes

2 No

8 I don't know/I don't remember

**Thank you for taking the time to fill out this questionnaire!**


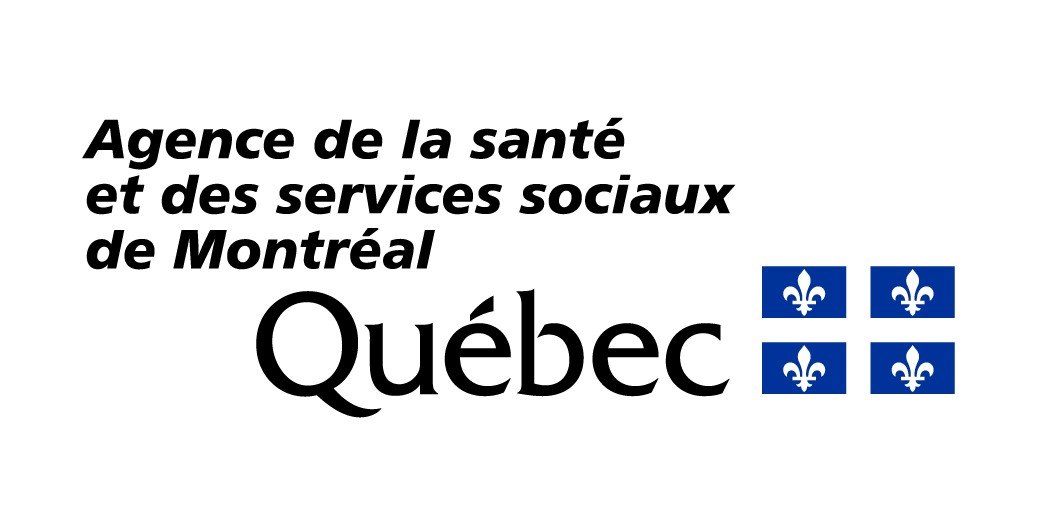


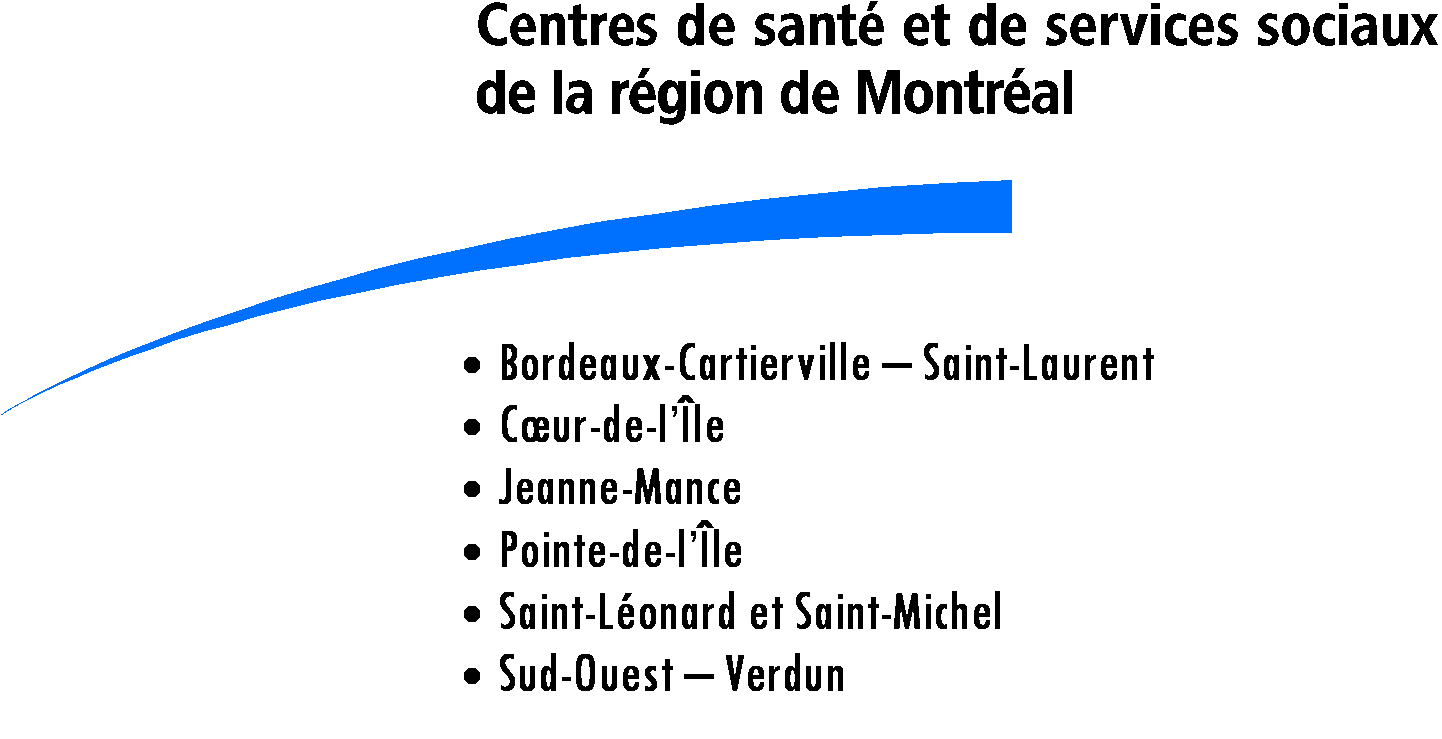

Supplement: Additional file 3 — Questionnaire for patients at 12, 24 and 36 months after entry into the program. This questionnaire includes questions on utilization of health services, experience of care with the primary care physician, management and follow-up of chronic illnesses, self-management and quality of life related to diabetes and/or hypertension. [file 1471-2296-12-126-S3.DOC]
