# Supplementary material for: Evaluation of the implementation of an integrated primary care network for prevention and management of cardiometabolic risk in Montréal
Source: BMC Fam Pract. 2011 Nov 10;12:126. doi: 10.1186/1471-2296-12-126 (PMC3282661; doi:10.1186/1471-2296-12-126)
Supplement: Additional file 4 — Questionnaire for patients who dropped out of the program during follow-up. Includes questions aimed at documenting the reasons for dropping out. [file 1471-2296-12-126-S4.DOC]

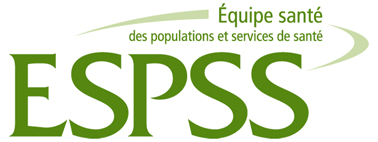

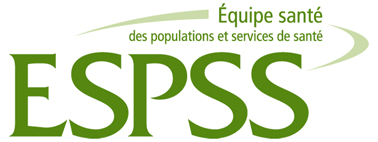
**Additional file 4**

File number: ___________ Site number: ___________

**Evaluation of the implementation of an integrated primary care network for prevention and management of cardiometabolic risk in Montréal**

**Questionnaire for patients**

**who drop out of the program during follow-up**

April 2011


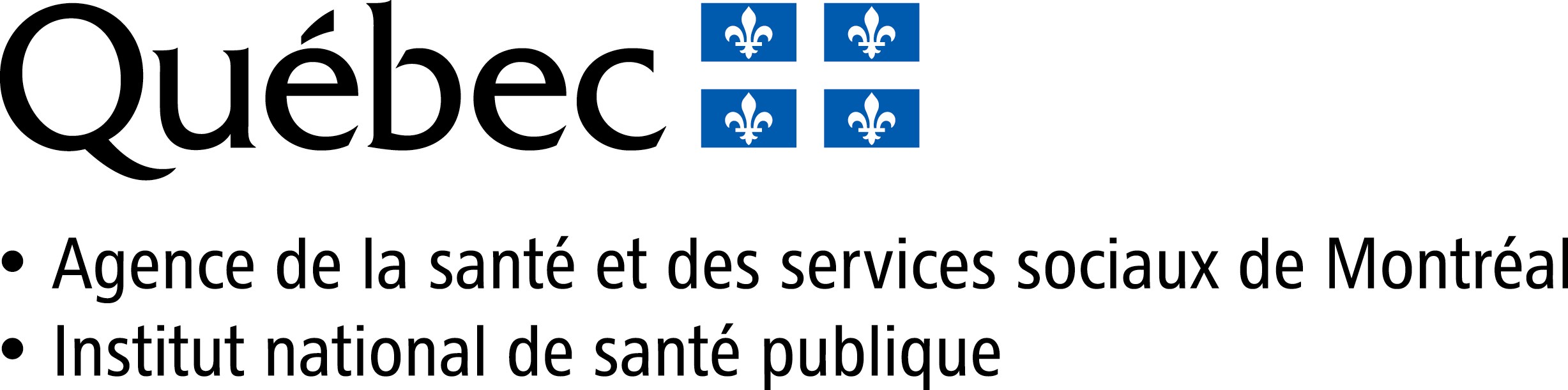


**Patients who drop out of the program after less than 12 months of follow-up are asked over the phone to answer the following question:**

We would like to know why you have dropped out of the CLSC’s program on changing lifestyle habits. Tell me whether or not each of the following statements applies to you.

|  |  | **Yes** | **No** | **Don't know/ Don't remember** |
| --- | --- | --- | --- | --- |
| AB1 | You moved | 1 | 2 | 8 |
| AB2 | You changed doctor and this doctor did not recommend that you participate in the CLSC’s program on changing lifestyle habits | 1 | 2 | 8 |
| AB3 | You lost your family doctor (e.g. the doctor has left the region, has retired or is on extended leave) | 1 | 2 | 8 |
|  |  |  |  |  |
| AB4 | At least one of the component of the CLSC’s program doesn't meet your needs. If so, which of the following components: | 1 | 2 | 8 |
|  | AB5 - the visits to the nurse | 1 | 2 | 8 |
|  | AB6 - the visits to the nutritionist | 1 | 2 | 8 |
|  | AB7 - groups sessions | 1 | 2 | 8 |
|  | AB8 - the physical activity program | 1 | 2 | 8 |
|  |  |  |  |  |
| AB9 | The CLSC’s program on changing lifestyle habits is too demanding | 1 | 2 | 8 |
| AB10 | The CLSC’s program requires too much time | 1 | 2 | 8 |
| AB11 | The schedule of the CLSC’s program doesn't suit you | 1 | 2 | 8 |
|  |  |  |  |  |
| AB12 | You consider that you don’t need this kind of service anymore (the first visits were sufficient) | 1 | 2 | 8 |
| AB13 | You consider that your diabetes or high blood pressure is not severe enough to participate in the CLSC’s program | 1 | 2 | 8 |
| AB14 | You consider that having your doctor monitor your diabetes or high blood pressure is enough | 1 | 2 | 8 |
| AB15 | You are already being followed elsewhere in a similar program | 1 | 2 | 8 |
| AB16 | You are afraid that if you participate in the program, you will be identified as diabetic or hypertensive, and this will affect your insurances or your job | 1 | 2 | 8 |
|  |  |  |  |  |
| AB17 | Your health has deteriorated too much to allow you to participate in the CLSC’s program on changing lifestyle habits | 1 | 2 | 8 |
| AB18 | Other reason *Specify*:_________________________________________________________________ __________________________________________________________________________________ | | | |

**Thank you for taking the time to answer this questionnaire!**
