# Supplementary material for: Evaluation of the implementation of an integrated primary care network for prevention and management of cardiometabolic risk in Montréal
Source: BMC Fam Pract. 2011 Nov 10;12:126. doi: 10.1186/1471-2296-12-126 (PMC3282661; doi:10.1186/1471-2296-12-126)

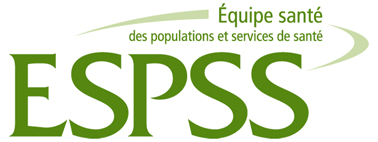
**Additional file 5**

Questionnaire no. ________

**Evaluation of the implementation of an integrated primary care network for prevention and management of cardiometabolic risk in Montréal**

**Questionnaire for Primary Care Physicians**

**at registration of a 1^st^ patient to the cardiometabolic risk program**

March 2011


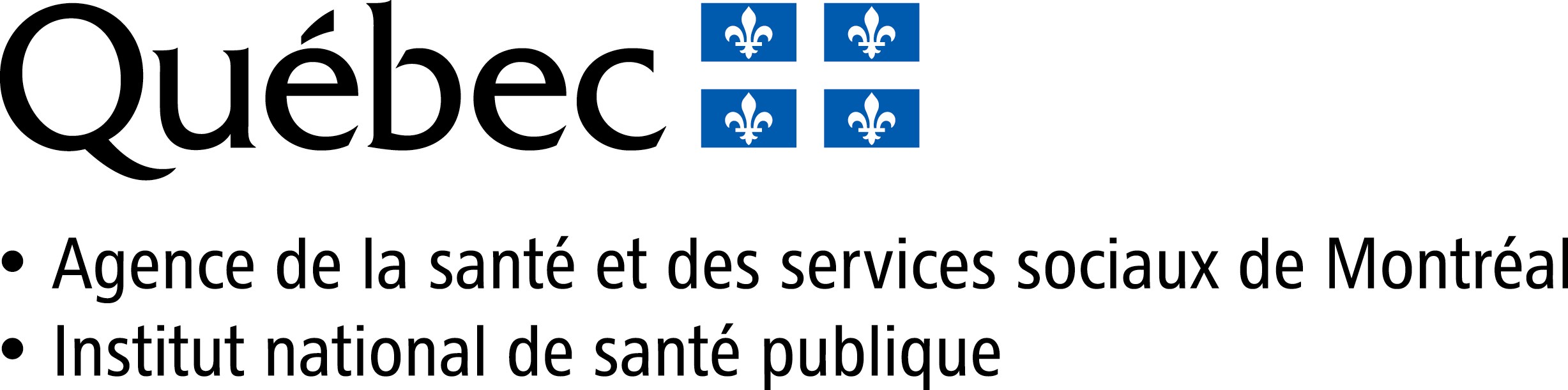


Dear Doctor,

Over the past few weeks, you have referred patients to the CSSS's cardiometabolic risk management and prevention program (on changing lifestyle habits).

We would like to know the characteristics of the primary care clinics participating in the cardiometabolic risk program implemented by the CSSS.

*Thank you for answering our questionnaire!*

| **Section A: Organizational Characteristics of Your Clinic** |
| --- |

1. **What type of primary care clinic is the clinic where you see most of the patients with diabetes or hypertension that you refer to the cardiometabolic risk program set up by the CSSS?**

1 Medical clinic or private office

2 Primary care clinic (adult) in a CLSC

3 FMU

4 Network clinic

5 Other *Specify:* __________________________________________

1. **Is this clinic an FMG?**

1 Yes  2  No

*The following questions are about this clinic.*

1. **In all, how many general practitioners currently work at the clinic** (including physicians working part time)**?** ______
2. **How many nurses currently work at the clinic** (including nurses working part time)**?**  ______

*(Note: in a CLSC, consider only nurses working in the adult primary care clinic)*

1. **Do general practitioners in your clinic share patient management?**

1 Yes  2  No  3  Doesn’t apply because only one doctor in the clinic

1. **What percentage of consultations do walk-in visits represent at your clinic?**

1 0%  2 1% to 25%  3 26% to 50%  4 51% to 75%  5 76% to 100%

1. **In your clinic, do you use …**
2. computer software to manage appointments?  1 Yes  2  No
3. Internet access for physicians? _1_ Yes  2  No
4. access to the health and social services telecommunications network (RTSS)? _1_ Yes  2  No
5. electronic medical records?  1 Yes  2  No
6. a Web-based appointment system for patients? _1_ Yes  2  No
7. an electronic interface to laboratory services or diagnostic imaging?  1 Yes  2  No
8. an electronic system to transmit prescriptions to pharmacies? _1_ Yes  2  No
9. computerized tools to aid medical decision-making
   (computerized reminders, automated alerts)?  1 Yes  2  No
10. computerized continuing professional development tools?  1 Yes  2  No
11. clinical practice guidelines integrated with electronic medical records?  1 Yes  2  No
12. others 🢂 *Specify:* __________________________________________  1 Yes  2  No
13. **In your clinic, do you have**…

|  | **No** | **Yes** | |
| --- | --- | --- | --- |
|  |  | **Computerized** | **Paper** |
| - 1. a reminder system to prompt patients to have the recommended screening tests (e.g. Pap test)? | 3 | 1 | 2 |
| - 1. a checklist in patient files concerning the preventive clinical practices (counselling, screening, immunization) to carry out with patients, based on current guidelines? | 3 | 1 | 2 |
| - 1. tools to assist lifestyle habit counselling (e.g. for smoking cessation interventions)? | 3 | 1 | 2 |
| - 1. tools to refer patients to services offering support for lifestyle changes (e.g. smoking cessation centre, health education centre)? | 3 | 1 | 2 |

1. **What are the roles and functions of the nurses on your medical team?** Check all that apply

*(Note: in a CLSC, consider only nurses working in the adult primary care clinic)*

1 There's no nurse on our team

2 Triage of walk-in patients

3 Counselling on tobacco use, diet and physical activity

4 Health education for patients (e.g. blood glucose testing, blood pressure measurement)

5 Systematic follow-up of specific clienteles

6 Liaison and coordination with CLSC, long-term care facilities, hospital centres and other clinics

7 Support for physicians' clinical activities (e.g. blood pressure measurement, weight, injections)

8 Participation in clinical decisions

9 Conducting clinical activities as part of a collective prescription

1. **In the building in which your clinic is located, are blood testing services available on-site?**

1 Yes  2  No

1. **In the building in which your clinic is located,**
2. are there any medical specialists?

1 Yes 🢂 If yes, what types of specialists are they? **__**__________________________________________________

____________________________________________________

2  No 🢂 Go to question 12 ____________________________________________________

1. To what degree do you collaborate (discussions, communications, referrals) with the medical specialists who are IN THE SAME BUILDING as your clinic?

1 A lot  2 Somewhat  3 Not much  4 Not at all

1. **In the building in which your clinic is located,…**
   1. are there any health professionals other than physicians?

1 Yes 🢂 If yes, what types of professionals are they? **_**___________________________________________________

____________________________________________________

2  No 🢂 Go to question 13 ____________________________________________________

- 1. To what degree do you collaborate (discussions, referrals) with the health professionals other than physicians who are IN THE SAME BUILDING as your clinic?

1 A lot  2 Somewhat  3 Not much  4 Not at all

1. **In your clinic, is there an on-call system outside opening hours for patients with chronic diseases (e.g. diabetes, hypertension) who have a family doctor at your clinic?**

1 Yes  2  No

1. **Is it possible for patients with chronic diseases (e.g. diabetes, hypertension) who have a family doctor at your clinic to contact a physician or a nurse by telephone when the clinic is open?**

1 Yes  2  No

| **Section B: Your Primary Care Practice** |
| --- |

*The following questions refer to your personal practice in the clinic where you see most of the patients with diabetes or hypertension that you refer to the CSSS’s cardiometabolic risk program.*

1. **In your own primary care practice, what percentage of consultations do walk-in visits represent?**

1 0%  2 1% to 25%  3 26% to 50%  4 51% to 75%  5 76% to 100%

1. **On average, how many patients do you see in your primary care clinic (including visits by appointment and walk-in visits) in a regular week of work?** _________
2. **On a typical day of primary care visits by appointment, what percentage do patients with diabetes and/or hypertension represent?**

1 Less than 10%  2 10% to 19%  3 20% to 29%  4 30% to 39%  5 40% to 49%  6 50% or over

1. **How long have you been referring patients to the diabetes or hypertension program at the CSSS? ______________**
2. **How were you informed about the CSSS cardiometabolic risk (diabetes and hypertension) program?**

1 At a CME session

2  By the CSSS (letter, flyer, …)

3  By colleagues

4  By a pharmaceutical representative

5  Other 🢂 *Specify:* __________________________________________

1. **In your own primary care practice, for follow-up of patients with diabetes or hypertension, you...**

|  | **Always** | **Usually** | **Occasionally** | **Rarely** | **Never** |
| --- | --- | --- | --- | --- | --- |
| 1. use a registry to identify and/or track patient care | 1 | 2 | 3 | 4 | 5 |
| 1. use a tracking system to remind patients about needed visits or services | 1 | 2 | 3 | 4 | 5 |
| 1. follow up patients by telephone between clinic visits (by the physician or clinic staff) | 1 | 2 | 3 | 4 | 5 |
| 1. use published practice guidelines as the basis for their treatment plan | 1 | 2 | 3 | 4 | 5 |
| 1. involve office staff (administrative or clerical) in identifying and reminding patients in need of follow-up or other services | 1 | 2 | 3 | 4 | 5 |
| 1. assist patients in setting and attaining self-management goals (e.g. patient participation in their own care management) | 1 | 2 | 3 | 4 | 5 |
| 1. refer patients to someone **within your clinic** for education about their chronic illnesses | 1 | 2 | 3 | 4 | 5 |
| 1. refer patients to someone **outside your clinic** for education about their chronic illnesses | 1 | 2 | 3 | 4 | 5 |
| 1. use flow sheets in medical files to track critical elements of care listed in patient management guidelines (e.g. glycated HB in diabetic) | 1 | 2 | 3 | 4 | 5 |

| **Section C: General Information** |
| --- |

The following data are essential to analyze your responses. Rest assured that these data will be kept *anonymous and confidential*.

1. **Are you:**  1 a man  2  a woman
2. **In what year did you obtain your medical diploma (MD)?** _________
3. **What percentage of your professional activities takes place in the following practice settings?**

| 1. Primary care clinic (in a medical clinic, private office, CLSC or FMU), whether or not it is an FMG | ______ % |
| --- | --- |
| 1. CLSC (home care) | ______ % |
| 1. CLSC (services other than home care and primary care) | ______ % |
| 1. Emergency department at a general and specialized hospital centre (CHSGS) | ______ % |
| 1. Short-term care unit at a general and specialized hospital centre | ______ % |
| 1. Long-term care facility (CHSLD) | ______ % |
| 1. Palliative care program | ______ % |
| 1. Obstetrics department in a hospital centre | ______ % |
| 1. Other 🢂 *Specify:* _______________________________________ | ______ % |
| Total | 100% |

THANK YOU FOR YOUR COOPERATION!

If you have any additional comments, please write them down in the space provided below.

We will read them very attentively.

_________________________________________________________________________________________________________

_________________________________________________________________________________________________________

_________________________________________________________________________________________________________

_________________________________________________________________________________________________________

_________________________________________________________________________________________________________

_________________________________________________________________________________________________________

_________________________________________________________________________________________________________

_________________________________________________________________________________________________________


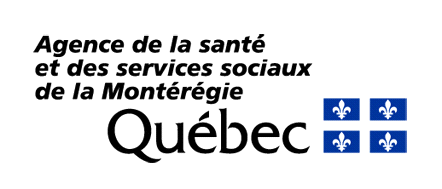

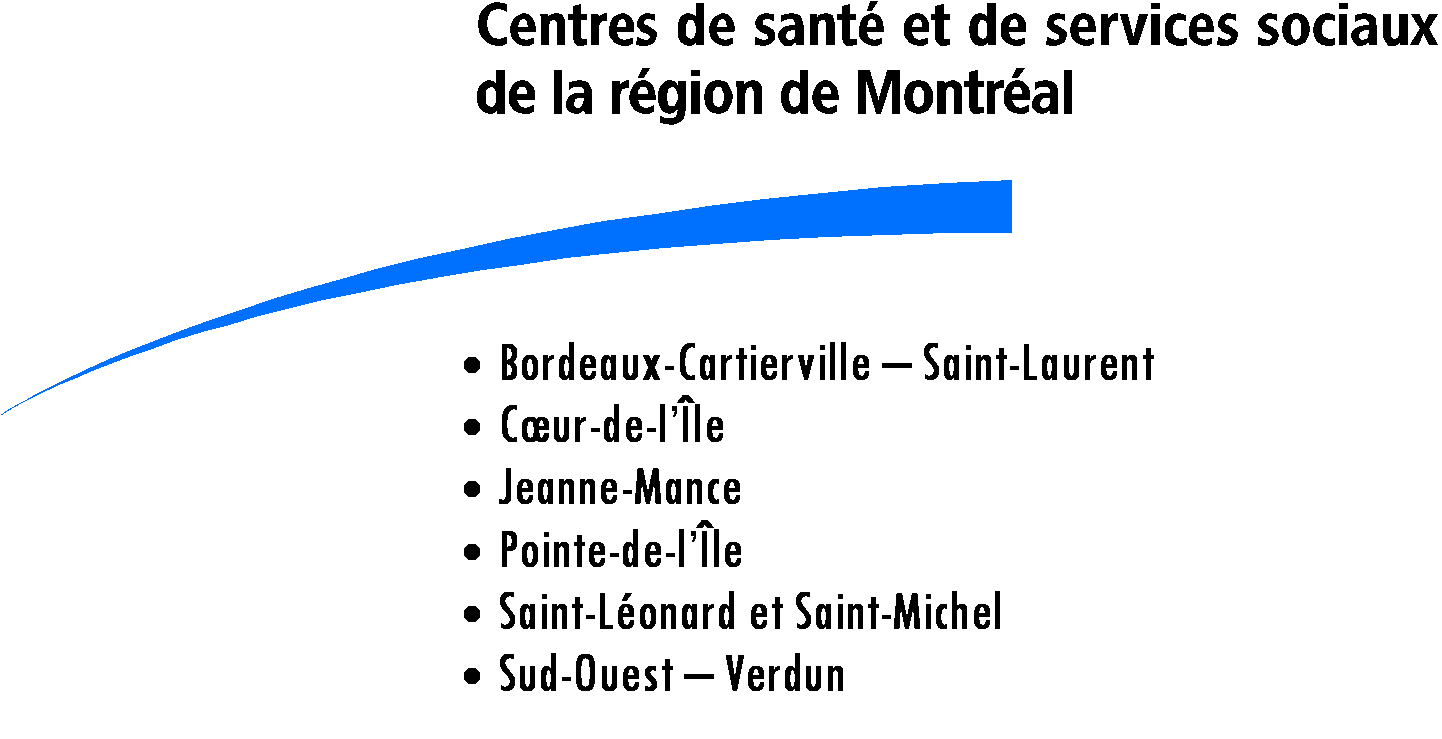

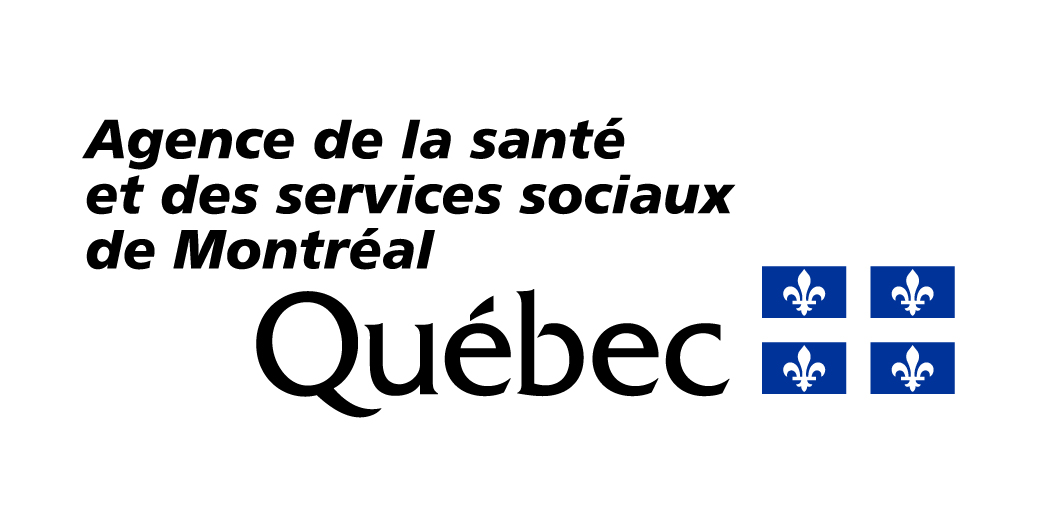

Supplement: Additional file 5 — Questionnaire for primary care physicians at registration of a 1st patient to the cardiometabolic risk program. This questionnaire contains questions pertaining to physicians' sociodemographic and professional characteristics, to the organizational characteristics of their clinics and to the physicians' practices regarding management of patients with diabetes or hypertension. [file 1471-2296-12-126-S5.DOC]
