# Supplementary material for: Evaluation of the implementation of an integrated primary care network for prevention and management of cardiometabolic risk in Montréal
Source: BMC Fam Pract. 2011 Nov 10;12:126. doi: 10.1186/1471-2296-12-126 (PMC3282661; doi:10.1186/1471-2296-12-126)

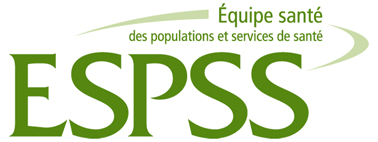
Additional file 6

Questionnaire no. ________

**Evaluation of the implementation of an integrated primary care network for prevention and management of cardiometabolic risk in Montréal**

**Questionnaire for Primary Care Physicians**

**12-24-36 months after registration of a 1^st^ patient
to the cardiometabolic risk program**

April 2011


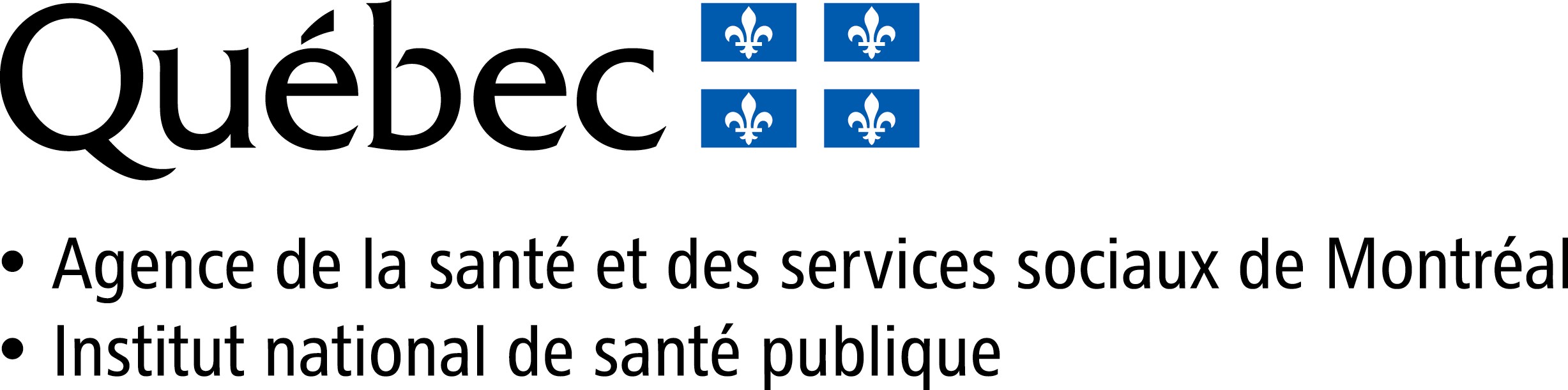


*Dear Doctor,*

*Over the past few months, you have referred patients to the CSSS's cardiometabolic risk management and prevention program (on changing lifestyle habits).*

*We would like to have your opinion about the program implemented by the CSSS. We would also like to ask you about the characteristics of your practice with diabetic and hypertensive patients.*

*Thank you for answering our questionnaire!*

| **Section A: Your primary care practice** |
| --- |

1. **In the past year, about how many patients with diabetes and/or hypertension did you refer to the cardiometabolic risk education program set up by the CSSS? _______**
2. **Do you continue to refer patients to the cardiometabolic risk program?**

1 Yes  2  No

1. **On a typical day of primary care visits by appointment, what percentage do patients with diabetes and/or hypertension represent?**

1 Less than 10%  2 10% to 19%  3 20% to 29%  4 30% to 39%  5 40% to 49%  6 50% or over

1. **In your own primary care practice, for follow-up of patients with diabetes or hypertension, you...**

|  | **Always** | **Usually** | **Occasionally** | **Rarely** | **Never** |
| --- | --- | --- | --- | --- | --- |
| 1. use a registry to identify and/or track patient care | 1 | 2 | 3 | 4 | 5 |
| 1. use a tracking system to remind patients about needed visits or services | 1 | 2 | 3 | 4 | 5 |
| 1. follow up patients by telephone between clinic visits (by the physician or clinic staff) | 1 | 2 | 3 | 4 | 5 |
| 1. use published practice guidelines as the basis for their treatment plan | 1 | 2 | 3 | 4 | 5 |
| 1. involve office staff (administrative or clerical) in identifying and reminding patients in need of follow-up or other services | 1 | 2 | 3 | 4 | 5 |
| 1. assist patients in setting and attaining self-management goals (e.g. patient participation in their own care management) | 1 | 2 | 3 | 4 | 5 |
| 1. refer patients to someone **within your clinic** for education about their chronic diseases | 1 | 2 | 3 | 4 | 5 |
| 1. refer patients to someone **outside your clinic** for education about their chronic diseases | 1 | 2 | 3 | 4 | 5 |
| 1. use flow sheets in medical files to track critical elements of care listed in patient management guidelines (e.g. glycated HB in diabetics) | 1 | 2 | 3 | 4 | 5 |

| **Section B: Your Opinion on the Cardiometabolic Risk Management and Prevention Program (on changing lifestyle habits)** |
| --- |

**Impact of the program on your patients**

1. **Indicate whether you totally or partly agree or partly or totally disagree with the following statements.**

| Most patients you have referred to the cardiometabolic risk program implemented by the CSSS…  (compared with their condition before their entry into the program) | **Totally  agree** | **Partly agree** | **Partly disagree** | **Totally**  **disagree** |
| --- | --- | --- | --- | --- |
| 1. control their diabetes or hypertension better | 1 | 2 | 3 | 4 |
| 1. comply with their medication better | 1 | 2 | 3 | 4 |
| 1. are better informed about their disease | 1 | 2 | 3 | 4 |
| 1. are more motivated to control their disease | 1 | 2 | 3 | 4 |
| 1. have improved their lifestyle habits | 1 | 2 | 3 | 4 |
| 1. are more able to self-manage their disease | 1 | 2 | 3 | 4 |
| 1. go less often to emergency for conditions associated with their diabetes or hypertension | 1 | 2 | 3 | 4 |
| 1. are hospitalized less often for conditions associated with their diabetes or hypertension | 1 | 2 | 3 | 4 |

**Impact of the program on your practice**

1. **Indicate whether you totally or partly agree or partly or totally disagree with the following statements.**

| The cardiometabolic risk program implemented by the CSSS… | **Totally  agree** | **Partly agree** | **Partly disagree** | **Totally**  **disagree** |
| --- | --- | --- | --- | --- |
| 1. has provided continuing professional development activities that are useful for your practice | 1 | 2 | 3 | 4 |
| 1. has provided clinical tools (e.g. guideline summaries, documents for patients) that are useful for your practice | 1 | 2 | 3 | 4 |
| 1. has enabled you to improve your knowledge about management of patients with diabetes or hypertension | 1 | 2 | 3 | 4 |
| 1. has enabled you to improve your knowledge about resources available for your patients with diabetes or hypertension | 1 | 2 | 3 | 4 |
| 1. is a useful addition to your practice | 1 | 2 | 3 | 4 |
| 1. reduces the time spent teaching these patients | 1 | 2 | 3 | 4 |
| 1. helps ensure better patient follow-up | 1 | 2 | 3 | 4 |

**Your relationship with the CSSS’s cardiometabolic risk program**

1. **Indicate whether you totally or partly agree or partly or totally disagree with the following statements.**

|  | **Totally  agree** | **Partly agree** | **Partly disagree** | **Totally**  **disagree** |
| --- | --- | --- | --- | --- |
| 1. Most of your patients with diabetes or hypertension meet the program's eligibility criteria | 1 | 2 | 3 | 4 |
| 1. You refer most of your eligible patients with diabetes or hypertension to the program | 1 | 2 | 3 | 4 |
| 1. Most patients with diabetes or hypertension that you refer to the program are seen within a reasonable period of time | 1 | 2 | 3 | 4 |
| 1. You get clinical feedback about all patients you refer to the program | 1 | 2 | 3 | 4 |

**Your relationship with specialized services at the CSSS**

1. **Indicate whether you totally or partly agree or partly or totally disagree with the following statements.**

| The cardiometabolic risk program implemented by the CSSS… | **Totally  agree** | **Partly agree** | **Partly disagree** | **Totally**  **disagree** |
| --- | --- | --- | --- | --- |
| 1. enables you to refer your patients with diabetes or hypertension to specialists more easily | 1 | 2 | 3 | 4 |
| 1. ensures you get better clinical feedback from the specialists consulted | 1 | 2 | 3 | 4 |
| 1. has set up a basis for collaboration with the CSSS for your other patients | 1 | 2 | 3 | 4 |

**Impact of the cardiometabolic risk program on collaboration in the CSSS territory**

1. **Indicate whether you totally or partly agree or partly or totally disagree with the following statements.**

| The cardiometabolic risk program implemented by the CSSS… | **Totally  agree** | **Partly agree** | **Partly disagree** | **Totally**  **disagree** |
| --- | --- | --- | --- | --- |
| 1. has generally increased collaborations between the CSSS, your clinic and other resources in the local services network | 1 | 2 | 3 | 4 |
| 1. has given rise to the creation of other chronic disease management networks | 1 | 2 | 3 | 4 |
| 1. has consolidated networking in the CSSS territory | 1 | 2 | 3 | 4 |

**Overall evaluation of the cardiometabolic risk management and prevention program**

1. In your opinion, has the program improved quality of care for patients with diabetes or hypertension?

________________________________________________________________________________________________________

________________________________________________________________________________________________________

________________________________________________________________________________________________________

________________________________________________________________________________________________________

1. In your opinion, what are the overall strengths and weaknesses of this program?

________________________________________________________________________________________________________

________________________________________________________________________________________________________

________________________________________________________________________________________________________

________________________________________________________________________________________________________

1. In your opinion, what factors have facilitated implementation of the program in your CSSS territory?

________________________________________________________________________________________________________

________________________________________________________________________________________________________

________________________________________________________________________________________________________

________________________________________________________________________________________________________

1. In your opinion, what factors have hampered implementation of the program in your CSSS territory?

________________________________________________________________________________________________________

________________________________________________________________________________________________________

________________________________________________________________________________________________________

________________________________________________________________________________________________________

THANK YOU FOR YOUR COOPERATION!

If you have any additional comments, please write them down in the space provided below.

We will read them very attentively.

___________________________________________________________________________________________________________

___________________________________________________________________________________________________________

___________________________________________________________________________________________________________

___________________________________________________________________________________________________________


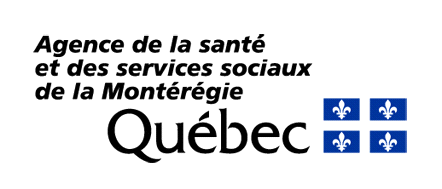

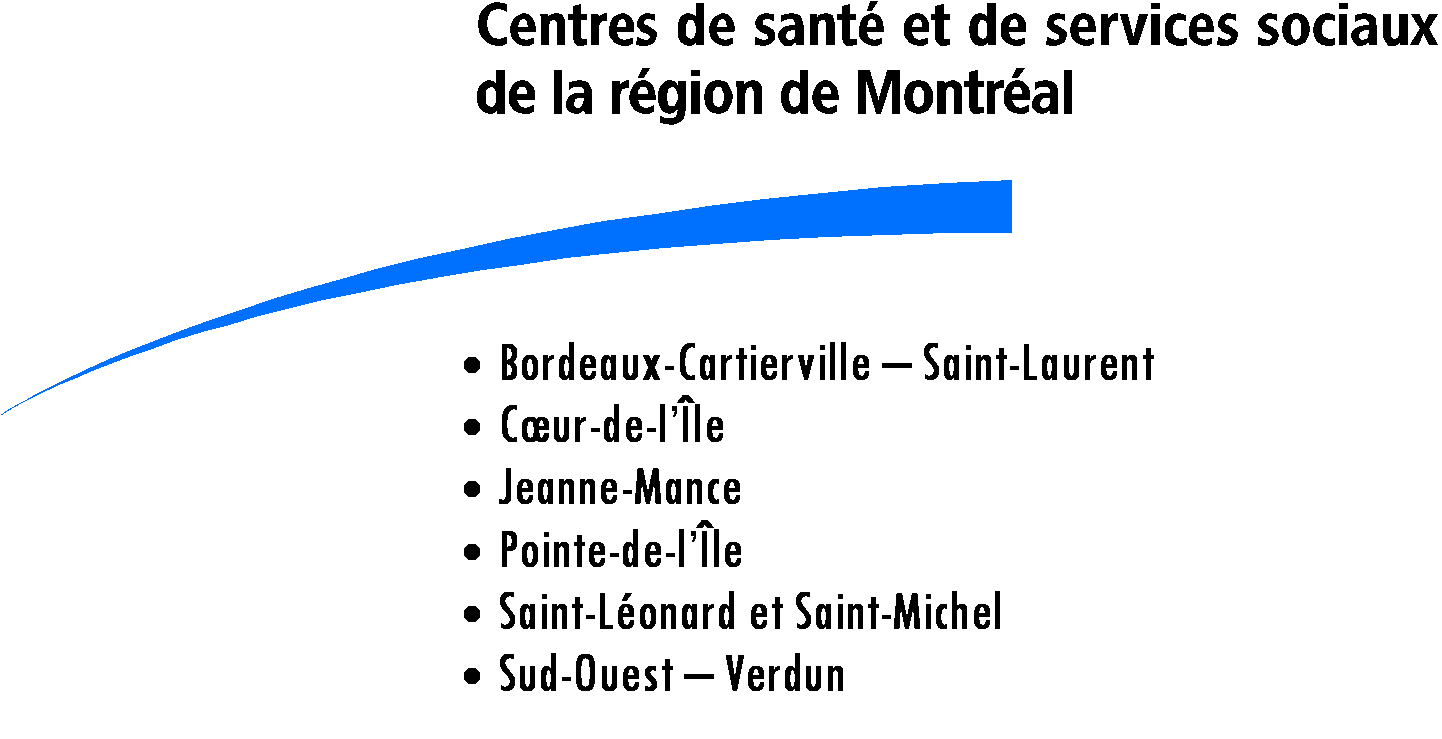

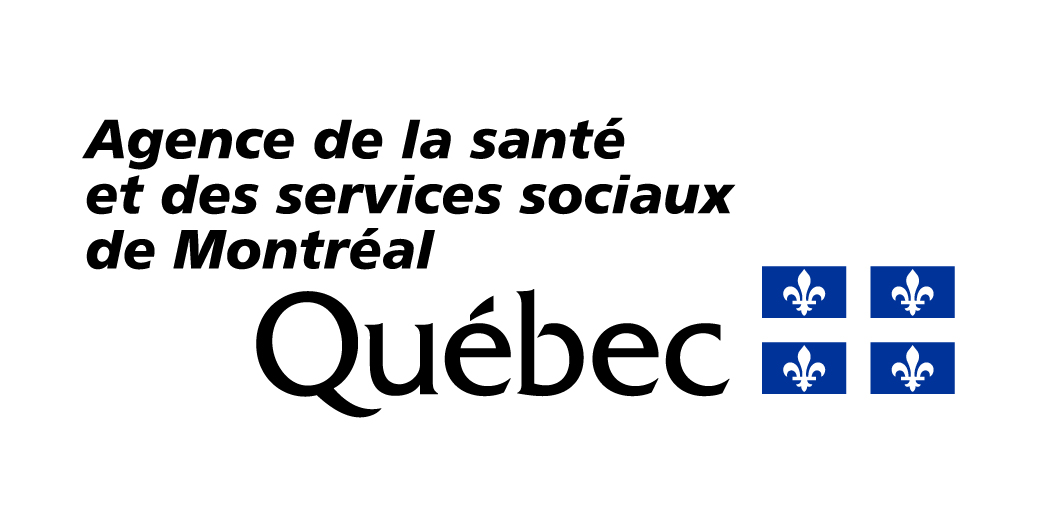

Supplement: Additional file 6 — Questionnaire for primary care physicians 12-24-36 months after registration of a 1st patient to the cardiometabolic risk program. This questionnaire includes questions on their assessment of the program: participation in the program, impacts on their patients and on their practice, relationships with specialized services, strengths and weaknesses of the program, as well as barriers and facilitating factors. [file 1471-2296-12-126-S6.DOC]
