# Supplementary material for: Evaluation of the implementation of an integrated primary care network for prevention and management of cardiometabolic risk in Montréal
Source: BMC Fam Pract. 2011 Nov 10;12:126. doi: 10.1186/1471-2296-12-126 (PMC3282661; doi:10.1186/1471-2296-12-126)
Supplement: Additional files 7 — Interview guide with individuals in charge of program implementation. These files contain discussion themes for the semi-structured interviews conducted at the beginning of the project, with individuals from the ASSS and CSSS who are responsible for program implementation. Discussion themes include an initial portrait of the services organization related to the activities of the program being implemented, the program components (objectives and purposes, role and actors involved, resources and activities), the dynamics in each implementation site. [file 1471-2296-12-126-S7.DOC]

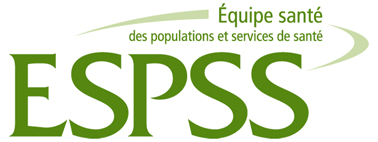
Additional file 7a

**Evaluation of the implementation of an integrated primary care network for prevention and management of cardiometabolic risk in Montréal**

**Interview guide**

**Agence de la santé et des services sociaux de Montréal (ASSSM)**

**Regional respondents**

**T0 (February 2011)**

April 2011


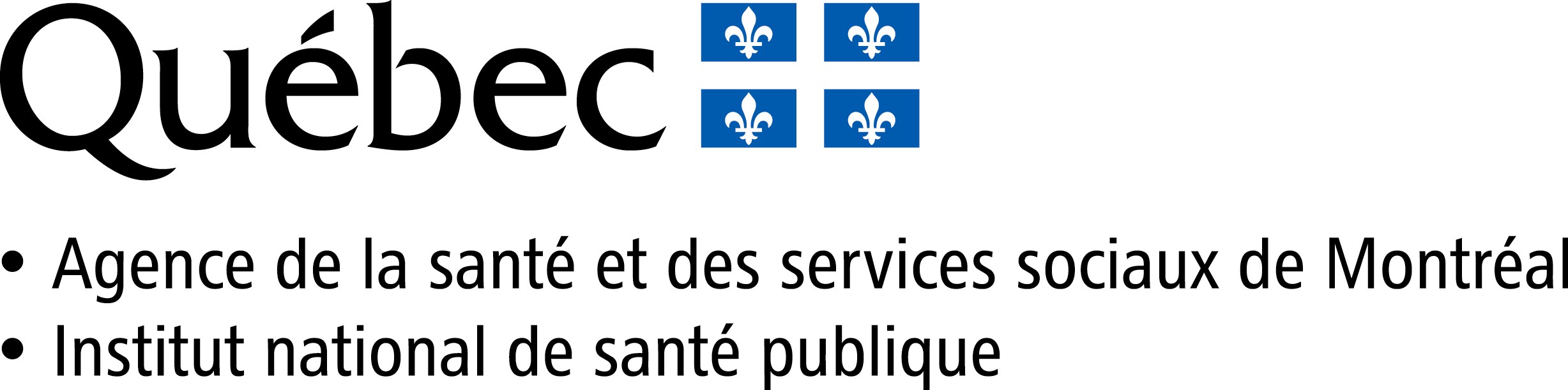


Detailed information about the Integrated and Interdisciplinary Cardiometabolic Risk Management and Prevention Program is available in documents provided by the ASSM. Interviews with regional respondents will provide additional information concerning the situation in Montréal and the ASSSM's involvement in program implementation by the CSSS.

| **Section A: Regional Context (historical and organizational)** |
| --- |

**Regional context related to services for people with diabetes/hypertension/cardiometabolic risk (CMR)**

1. Overall in Montréal, how are services for people with diabetes and hypertension organized (e.g. specialized care networks/service corridors and agreements/actors involved/sources of financing)?
2. What regional structures, resources and/or initiatives are already in place for diabetes and/or hypertension management (type and quantity)?
3. What role do these structures/resources play in the services offered to people with diabetes and/or hypertension?
4. What role does the ASSSM play in planning the implementation of services offered regionally?

**Context related to the diabetes and hypertension program**

1. Which regional organizational structures are directly or indirectly involved in the CMR program?

(e.g. regional committees, regional panels, hospital centres)

1. Which CSSS implemented the diabetes prevention and management program?
2. How does the ASSSM support CMR program implementation?
3. Can you estimate the current regional population coverage for this program?

| **Section B: Regional Leadership in the Implementation of the Cardiometabolic Risk Program** |
| --- |

1. To your knowledge, do one or several CSSS stand out because of their excellence or leadership in the organization of services related to diabetes or hypertension?
2. What role does the ASSSM play in implementing the program?

- Allocation of human and financial resources
- Technical support
- Development of an information system
- Development of a regional patient follow-up registry
- Others

1. What role does the ASSSM play in decision making related to local program implementation?
2. What role does the ASSSM play in developing common tools?
3. What role does the ASSSM play in standardizing practices?
4. What role does the ASSSM play in establishing and consolidating links with partners?
5. What role does the ASSSM play in program evaluation (e.g. developing performance indicators)?
6. How will the ASSSM use the evaluation results?

| **Section C: Climate in Which the Program Is Being Implemented** |
| --- |

1. Can you qualify the relationships related to this program?

- With participating CSSS
- With the Direction de santé publique
- With other ASSSM directorates
- With other partners and actors involved in the project (e.g. primary care clinics, hospitals)

1. Within the ASSSM, which financial and human resources are dedicated to implementing the program in the region?
2. What incentives and resources are allocated to the CSSS to foster program implementation?
3. Financial resources
4. Real estate resources
5. Material resources
6. Information resources
7. Planning tools for local managers
8. Practical tools (including educational tools for patients)
9. What do you expect participating CSSS will contribute (in relation to the incentives listed above and the resources required)?
10. What mechanisms have been put in place to ensure communication and feedback with CSSS?
11. What mechanisms have been put in place to ensure there is follow-up and that difficulties are identified and problems resolved?
12. What mechanisms have been put in place to ensure CSSS accountability?

| **Section D: Implementation of the Cardiometabolic Risk Program** |
| --- |

1. What responsibilities do CSSS have concerning program implementation in terms of
2. services offered (compliance with the intervention in its entirety)
3. support for primary care physicians and clinical settings
4. support for the interdisciplinary team from the teaching institution
5. What margin/latitude do local managers have to adapt the program based on local realities of the CSSS?

| **Section E: Determinants of Program Implementation** |
| --- |

1. In your opinion, what conditions or factors will contribute to implementation of the program in the CSSS territories?
2. In your opinion, what difficulties are likely to arise during implementation of the program in the CSSS territories?

| **Section F: Complementary Questionnaire – Vision and Values** |
| --- |

1. **In your opinion, to what degree will the cardiometabolic risk program help improve the following?**

|  | **A lot** | **Quite a bit** | **Little** | **Not at all** | **Don’t know** |
| --- | --- | --- | --- | --- | --- |
| 1. **Services offered** in your region for people with diabetes and/or hypertension |  |  |  |  |  |
| 1. **Quality of professional practices** related to services for people with diabetes and/or hypertension |  |  |  |  |  |
| 1. **Health of the population** with diabetes and/or hypertension |  |  |  |  |  |

1. **(A) How much importance do you give the following elements related to the cardiometabolic risk program?
   (B) How would you assess their feasibility?**

|  | **(A) Importance** | | | |  | **(B) Feasibility** | | |
| --- | --- | --- | --- | --- | --- | --- | --- | --- |
|  | **Very important** | **Quite important** | **Less important** | **Not important** |  | **Very feasible** | **Somewhat feasible** | **Not very feasible** |
| 1. Implementation of quality interventions |  |  |  |  |  |  |  |  |
| 1. Multidisciplinary approach |  |  |  |  |  |  |  |  |
| 1. Patient-centred approach |  |  |  |  |  |  |  |  |
| 1. Development of interventions targeting multiple morbidities |  |  |  |  |  |  |  |  |
| 1. Support of primary care physicians participating in the program |  |  |  |  |  |  |  |  |
| 1. Standardization of clinical practices and tools |  |  |  |  |  |  |  |  |
| 1. Implementation of an information-sharing system and creation of a computerized regional registry |  |  |  |  |  |  |  |  |
| 1. Systemization of communication procedures among service providers |  |  |  |  |  |  |  |  |
| 1. Approach fostering self-management and adoption of healthy behaviours |  |  |  |  |  |  |  |  |
| 1. Involvement of community resources |  |  |  |  |  |  |  |  |
| 1. Approach integrating lifestyle modification and prevention activities |  |  |  |  |  |  |  |  |
| 1. Establishment and consolidation of links with partners (e.g. networking, concertation table) |  |  |  |  |  |  |  |  |

1. **What are the regional organizational priorities related to implementation of the cardiometabolic risk program? Rate the following items by order of importance, with 1 being the most important and 6 the least important.**

|  | Improve access to chronic disease services for Montrealers who have diabetes or hypertension (increase population coverage) |
| --- | --- |
|  | Increase CSSS resources and staff |
|  | Extend the range of diabetes and hypertension services to the continuum of care (prevent–cure–support) |
|  | Standardize care trajectories for people with diabetes or hypertension |
|  | Develop links with primary care clinics in the territory and with CSSS partners |
|  | Improve quality of care for people with diabetes or hypertension |

| **Section G: General Information** |
| --- |

1. What is your profession?
2. What is your position at the Agence?
3. What is your role in the implementation of the cardiometabolic risk program?
4. How long have you had this role?

**Date: ________/_______/__________**

**(Day / Month / Year)**


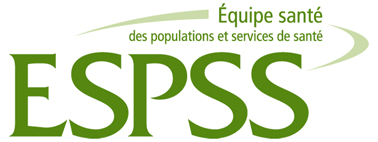
Additional file 7b

**Evaluation of the implementation of an integrated primary care network for prevention and management of cardiometabolic risk in Montréal**

**Interview guide**

**Local managers responsible for program implementation**

**T 0 (February 2011)**

April 2011

The objectives of collecting information from local managers through existing documentation and interviews are to

- draw up an initial profile of the services offered locally to people with diabetes and hypertension;
- document program activities already introduced and those to implement as well as allocated resources; and
- understand local dynamics

| **Section A: Local Context (historical and organizational)** |
| --- |

**General context related to services for people with diabetes/hypertension/cardiometabolic risk (CMR)**

1. Do you know what services are used by **people** living in your territory who have diabetes or hypertension, or by individuals with cardiometabolic risk (e.g. follow-up by family physicians in or outside the territory, in specialized clinics or in hospitals)

***The following questions concern services currently offered in your CSSS territory to people with diabetes or hypertension, or with cardiometabolic risk.***

1. What local structures, resources and/or initiatives are already in place for diabetes and/or hypertension management (type and quantity)?
2. What role do these structures/resources play in delivering services to people with diabetes and/or hypertension?
3. What role does the CSSS play in planning and/or implementing services offered in the territory?
4. Can you estimate the population coverage for the services and resources listed above among people with these diseases in your territory?

**Context related to the existing diabetes program – Clinical process**

1. a) What is the current composition of the interdisciplinary team that provides the program's clinical services?

b) How long has the program been running/has the team been seeing patients?

1. Describe the process used to refer primary care patients to the program:
   - Who refers patients? Who is the targeted clientele and what are the criteria (if applicable)?
   - Education centre referral methods and reception of new referrals
2. What clinical activities were conducted during the initial patient visit/evaluation?
3. Specify the clinical processes and activities related to monitoring biological parameters.
4. What process is used when referring patients to specialized services?
5. Specify the clinical process and activities related to group educational sessions.
6. Specify the clinical processes and activities related to individual follow-up by a nutritionist.
7. Specify the clinical processes and activities related to individual follow-up by a nurse.
8. Specify the clinical processes and activities related to the physical activity program.
9. What is the attending primary care physician's role in the clinical process?
10. How are clinical tasks shared (review of measurements/referrals to specialized care/lab readings) with the attending physician?
11. Specify the mechanisms in place to ensure feedback and knowledge transfer to the attending physician.
12. In terms of the diabetes program, have any elements or interventions been added to or withdrawn from the clinical process developed by the ASSSM?

If yes, what elements were adapted/changed?

**Context related to the existing diabetes program – Non-clinical process**

1. Has a coordination committee been set up?

If yes:

- 1. What is its mandate?
  2. Who is on it?

If no:

- 1. Are there plans to put together such a committee? Is there another coordinating structure?

1. Has a clinical committee been set up?

If yes:

- 1. What is its mandate?
  2. Who is on it?

If no:

- 1. Are there plans to put together such a committee? Is there another structure responsible for this?

1. What links have been made with outside partners (e.g. FMG and network clinics, health education centres, quit-smoking centres, pharmacies, community organizations, research centres) related to the diabetes program?
2. If applicable, indicate the liaison mechanisms set up with these external partners (e.g. planning/clinical coordination)
3. Specify the existing activities and processes in the current program that provide support to primary care physicians and the interdisciplinary team

**Context related to the existing diabetes program – Impact of the current program**

1. Can you estimate the program's population coverage for diabetes (number of patients seen and percentage of the population with diabetes seen in your territory)?
2. Can you estimate the number of doctors reached through the program (penetration rate in clinical settings)?
3. To date, what program advertising and promotion strategies have been used with clinics/primary care physicians?
4. How would you describe primary care physicians' commitment to the program in your territory?

| **Section B: Local leadership in Implementation of the Cardiometabolic Risk Program** |
| --- |

1. What role does the CSSS play in decision making related to program implementation?
   1. Allocation of human and financial resources
   2. Promotion and recruitment of physicians for referrals to the program
   3. Set up and consolidation of links with partners from the local services network
   4. Standardization of practices on the territory
   5. Development of a computerized system/technical support
   6. Development of common tools
   7. Program evaluation (e.g. development of performance indicators)
   8. Others (if applicable)
2. Who is the person in charge of implementing the program in your CSSS (name and position) ?
3. What are the roles and responsibilities of the following:
   1. Local manager
   2. Administrator in charge
   3. Coordinating committee
   4. Local clinical committee
4. What are the ASSSM's roles and responsibilities related to program implementation?
5. To your knowledge, do any clinical settings in your territory stand out because of their excellence or leadership in the organization of services for diabetes or hypertension?

| **Section C: Program Components to Deploy** |
| --- |

**Clinical process**

1. Does moving from a diabetes program to a broader cardiometabolic risk program (with implementation of the hypertension component) include adding new professionals to the interdisciplinary team?
2. Specify the activities as well as the clinical processes to put in place for the following elements of the hypertension component
3. Care trajectory (e.g. reception, individual and group meetings, referrals to external resources)
4. Reception of new patients referred
5. Support functions
   - 1. welcome and guidance
     2. appointment follow-up
6. Links with primary care physicians
7. Monitoring of biological parameters
8. Referrals to specialized services
9. Group educational sessions
10. Individual follow-up by a nutritionist
11. Individual follow-up by a nurse
12. Physical activity program
13. In terms of the activities in the broader cardiometabolic risk program (diabetes and hypertension) proposed by the ASSSM, do you plan on adding, changing or withdrawing any of the elements?

**Non-clinical processes (links among different partners and support for primary care physicians/interdisciplinary team)**

1. With the implementation of the hypertension component, do changes in the cardiometabolic risk program entail changes in the composition and/or mandate of local committees (coordination and clinical)? If yes, which ones?
2. With the implementation of the hypertension component, do changes in the cardiometabolic risk program entail changes in activities that support primary care physicians?
3. Do you intend to open up/promote the cardiometabolic risk program to all physicians in your territory, if this has not already been done?
   1. If yes, what promotional methods have been planned or implemented?
   2. If no, why not?
4. With the implementation of the hypertension component, do changes in the cardiometabolic risk program entail changes in activities that support the interdisciplinary team?
5. With the implementation of the hypertension component, do changes in the cardiometabolic risk program involve developing (or consolidating) new links and/or liaison mechanisms with external partners (hospitals/community organizations, associations, or others)?
6. What (if any) other administrative/non-clinical (e.g. weekly meetings) activities will be deployed to support and/or consolidate implementation of the hypertension component

- for the cardiometabolic risk education centre
- for primary care clinics
- for referrals to specialized care
- for follow-up with regional representatives

| **Section D: Program Implementation and Climate of Implementation** |
| --- |

1. Can you qualify relationships during the implementation of the cardiometabolic risk program with

- the ASSSM
- the evaluation team
- other partners and actors involved in the project (e.g. primary care clinics, hospitals)

1. Aside from the support activities already included in the diabetes/hypertension program, have any other mechanisms been planned to promote participation in the program and support primary care physicians?
   1. Knowledge transfer activities (workshops/lunchtime presentations/Web forum)
   2. Employees on loan
   3. Financial incentives
   4. Others
2. What incentives and CSSS resources have been allocated to program implementation?
3. Financial resources
4. Human resources (e.g. administrative officer)
5. Real estate resources
6. Material resources
7. Information resources
8. Clinical tools (including educational tools for patients)
9. How have these resources been allocated or incentives put in place (e.g. criteria, decision-making process)?
10. What is the regional contribution (of the ASSSM) to local program implementation (resources and incentives)?
11. Is there a framework document related to the program that healthcare professionals and/or local partners can consult?
12. Were the CSSS's policies and procedures modified in view of or during program implementation?
13. What mechanisms have been put in place to ensure communication and feedback among program partners?
14. What mechanisms have been put in place to ensure training and maintenance of clinical skills of professionals involved in the program?
15. What mechanisms have been put in place to ensure there is follow-up with local partners, and that difficulties are identified and problems resolved?

| **Section E: Determinants of Program Implementation** |
| --- |

1. In your opinion, which elements facilitated deployment of the diabetes program in your CSSS?
2. In your opinion, what conditions or factors would facilitate implementation of the cardiometabolic risk program (via addition of the hypertension component) in your CSSS territory?
3. In your opinion, which elements hindered deployment of the diabetes program in your CSSS?
4. In your opinion, what factors might work against program implementation in your CSSS territory?

| **Section F: Additional Questionnaire: Vision and Values** |
| --- |

1. **In your opinion, to what degree will the cardiometabolic risk program help improve the following?**

|  | **A lot** | **Quite a bit** | **Little** | **Not at all** | **Don’t know** |
| --- | --- | --- | --- | --- | --- |
| 1. **Services offered** in your territory for people with diabetes and/or hypertension |  |  |  |  |  |
| 1. **Quality of professional practices** related to services offered to people with diabetes and/or hypertension |  |  |  |  |  |
| 1. **Health of the population** with diabetes and/or hypertension |  |  |  |  |  |

1. **(A) How much importance do you give the following elements related to the cardiometabolic risk program? (B) In the context of your territory, how would you assess their feasibility?**

|  | **(A) Importance** | | | |  | **(B) Feasibility** | | |
| --- | --- | --- | --- | --- | --- | --- | --- | --- |
|  | **Very important** | **Quite important** | **Less important** | **Not important** |  | **Very feasible** | **Somewhat feasible** | **Not very feasible** |
| 1. Implementation of quality interventions |  |  |  |  |  |  |  |  |
| 1. Multidisciplinary approach |  |  |  |  |  |  |  |  |
| 1. Patient-centred approach |  |  |  |  |  |  |  |  |
| 1. Development of interventions targeting multiple morbidities |  |  |  |  |  |  |  |  |
| 1. Support of primary care physicians participating in the program |  |  |  |  |  |  |  |  |
| 1. Standardization of clinical practices and tools |  |  |  |  |  |  |  |  |
| 1. Implementation of an information-sharing system and creation of a computerized regional registry |  |  |  |  |  |  |  |  |
| 1. Systemization of communication procedures among service providers |  |  |  |  |  |  |  |  |
| 1. Approach fostering self-management and adoption of healthy behaviours |  |  |  |  |  |  |  |  |
| 1. Involvement of community resources |  |  |  |  |  |  |  |  |
| 1. Approach integrating lifestyle modification and prevention activities |  |  |  |  |  |  |  |  |
| 1. Establishment and consolidation of links with partners (e.g. networking, concertation table) |  |  |  |  |  |  |  |  |

1. **What are your CSSS's organizational priorities related to implementation of the cardiometabolic risk program? Rate the following items by order of importance, with 1 being the most important and 6 the least important.**

|  | Improve access to chronic disease services for people in the territory who have diabetes or hypertension (increase population coverage) |
| --- | --- |
|  | Increase CSSS resources and staff |
|  | Extend the range of diabetes and hypertension services to the continuum of care (prevent–cure–support) |
|  | Standardize care trajectories for people with diabetes or hypertension |
|  | Develop links with primary care clinics in the territory and with CSSS partners |
|  | Improve quality of care for people with diabetes or hypertension |

| **Section G: General Information** |
| --- |

1. What is your profession?
2. What is your job at the CSSS?
3. What is your role in the implementation of the cardiometabolic risk program?
4. How long have you had this role?

**Date: ________/_______/__________**

**(Day / Month / Year)**
