# Supplementary material for: Evaluation of the implementation of an integrated primary care network for prevention and management of cardiometabolic risk in Montréal
Source: BMC Fam Pract. 2011 Nov 10;12:126. doi: 10.1186/1471-2296-12-126 (PMC3282661; doi:10.1186/1471-2296-12-126)
Supplement: Additional file 8 — CSSS Questionnaire. This file contains the questionnaire used to document interorganizational collaboration within local services networks in a previous project [36]. [file 1471-2296-12-126-S8.DOC]

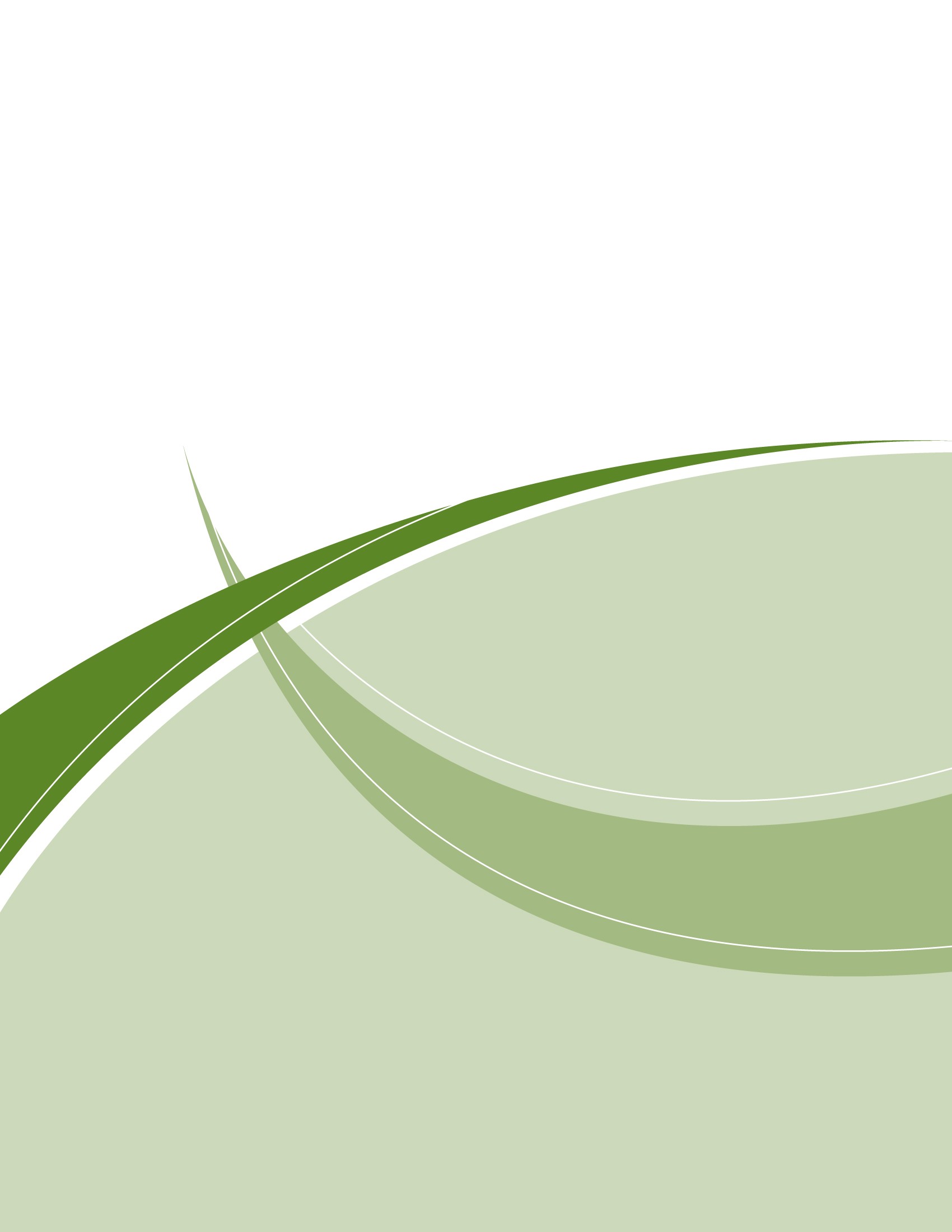
Additional file 8

**Assessing the evolution of primary healthcare organizations**

**and their performance (2005-2010) in two regions of**

**Québec province: Montréal and Montérégie**

**CSSS Questionnaire**

July 19, 2010

Institut national de santé publique du Québec

Direction de santé publique, Agence de la santé et des services sociaux de Montréal

**
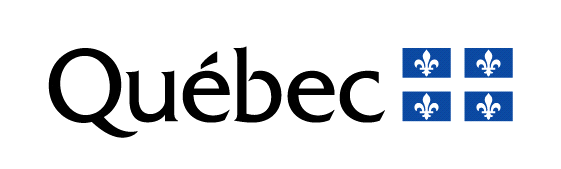
**

**Instructions**

**Preamble**

CSSS have a mandate to create local services networks and to implement new primary care organizational models (Family Medicine Groups, Network Clinics). Since their inception, CSSS have introduced changes to the health system. We would like you to tell us your assessment of these changes and their effects on primary care services, in particular on primary care organizations: medical clinics (solo, group, FMG) and other organizations that provide primary care (e.g. CLSC, FMU, health cooperatives, or others).

**In this questionnaire, the term “primary care services” refers to primary care medical services.**

**Who should answer this questionnaire?**

You have been identified, from among the managers in your CSSS, as the person who is best able to assess the recent changes brought to primary care organization.

**How should the questionnaire be completed?**

- The information and consent form that is integrated to the questionnaire has to be completed and signed
- Please answer to the best of your knowledge
- Circle or check off one answer per question, unless otherwise indicated

**Questionnaire components**

- This questionnaire includes 21 questions grouped into the following sections:

Section 1 – Collaboration between the CSSS and primary care organizations

Section 2 – Collaboration between primary care organizations and hospitals

Section 3 – Collaboration among primary care organizations

Section 4 – Collaboration of the CSSS with its partners

Section 5 – Support for the development of emerging primary care organizational models

Section 6 – Role of the Agence régionale and of the MSSS

Section 7 – Information about the respondent

- The questionnaire is also available online. Please refer to the letter included with this questionnaire (*www.greas.ca).*

If you have any questions, you can contact:

Dominique Grimard

Direction de santé publique

Agence de la santé et des services sociaux de Montréal

Équipe Santé des populations et services de santé

Telephone: (514) 528-2400, extension: 3563

**1 - Collaboration between the CSSS and primary care organizations**

1. Since your CSSS was set up, to what degree have the following mechanisms improved collaboration between your **CSSS** and the **primary care organizations** in its territory?

| **Mechanisms** | **A lot** | **Quite a lot** | **A little** | **Not at all** | **Was not implemented** |
| --- | --- | --- | --- | --- | --- |
| 1. Agreements between CSSS and medical clinics (e.g. planning services offered, opening hours) | 1 | 2 | 3 | 4 | 99 |
| 1. CSSS resources loaned to medical clinics | 1 | 2 | 3 | 4 | 99 |
| 1. Availability of CSSS resources for medical clinics (e.g. technical services) | 1 | 2 | 3 | 4 | 99 |
| 1. Others (specify): ____________________________   ____________________________ | 1 | 2 | 3 | 4 | 99 |

1. The following statements concern the mechanisms implemented to facilitate collaboration between your **CSSS** and the **primary care organizations** in its territory, identified in the preceding question. Indicate the degree to which you agree or disagree with each statement.

| Mechanisms… | **Strongly**  **agree** | **Agree** | **Disagree** | **Strongly**  **disagree** | Don't know/Not applicable |
| --- | --- | --- | --- | --- | --- |
| 1. benefit primary care clinics | 1 | 2 | 3 | 4 | 9 |
| 1. are somewhat onerous to manage | 1 | 2 | 3 | 4 | 9 |
| 1. help overcome obstacles to collaboration between various levels of care | 1 | 2 | 3 | 4 | 9 |
| 1. foster development of service corridors with secondary and tertiary care institutions | 1 | 2 | 3 | 4 | 9 |
| 1. act as a leverage to implement changes in professional practices | 1 | 2 | 3 | 4 | 9 |
| 1. foster better coordination of primary care services | 1 | 2 | 3 | 4 | 9 |
| 1. help increase beneficiaries' awareness of the services available in the territory | 1 | 2 | 3 | 4 | 9 |

1. Overall, to what degree has your CSSS supported and encouraged collaboration between your **CSSS (excluding the hospital component)** and the **primary care organizations** in its territory?

1 A lot  2 Quite a lot  3 A little  4 Not at all

**2 - Collaboration between primary care organizations and hospitals**

1. Since your CSSS was set up, to what degree have the following mechanisms improved collaboration between **primary care organizations** and the **hospitals** in its territory?

| **Mechanisms** | **A lot** | **Quite a lot** | **A little** | **Not at all** | **Was not implemented** |
| --- | --- | --- | --- | --- | --- |
| 1. Service agreement involving the CSSS, hospitals and medical clinics | 1 | 2 | 3 | 4 | 99 |
| 1. Coordination/concertation tables that include representatives from hospitals and medical clinics from the territory | 1 | 2 | 3 | 4 | 99 |
| 1. Development of a joint clinical project | 1 | 2 | 3 | 4 | 99 |
| 1. Others (specify): _____________________________   _____________________________ | 1 | 2 | 3 | 4 | 99 |

1. Overall, to what degree has your CSSS supported and encouraged collaboration between **primary care organizations** and **hospitals** in its territory (including the CSSS hospital centre, if applicable)?

1 A lot  2 Quite a lot  3 A little  4 Not at all

**3 - Collaboration among primary care organizations**

1. Since your CSSS was set up, to what degree have the following mechanisms improved collaboration among **primary care organizations** in its territory?

| **Mechanisms** | **A lot** | **Quite a lot** | **A little** | **Not at all** | **Was not implemented** |
| --- | --- | --- | --- | --- | --- |
| 1. Primary care services coordination/concertation tables | 1 | 2 | 3 | 4 | 99 |
| 1. Physicians' participation in planning primary care services available in the territory | 1 | 2 | 3 | 4 | 99 |
| 1. Training offered by the CSSS to physicians in the territory (continuing clinical and medical-administrative education ) | 1 | 2 | 3 | 4 | 99 |
| 1. Local DRMG's participation in the CSSS's managing team | 1 | 2 | 3 | 4 | 99 |
| 1. Implementation of an "entry point" for vulnerable orphan patients | 1 | 2 | 3 | 4 | 99 |
| 1. Clinical reception for subacute cases | 1 | 2 | 3 | 4 | 99 |
| 1. Others (specify): _____________________________   _____________________________ | 1 | 2 | 3 | 4 | 99 |

1. Overall, to what degree has your CSSS supported and encouraged collaboration among the various **primary care organizations** in its territory?

1 A lot  2 Quite a lot  3 A little  4 Not at all

**4 - Collaboration of the CSSS with its partners**

1. To date, how would you qualify your CSSS's collaboration with the **following partners**?

|  | **Very good** | **Good** | **Poor** | **None** | Don't know/ **Not applicable** |
| --- | --- | --- | --- | --- | --- |
| 1. Primary healthcare clinics | 1 | 2 | 3 | 4 | 9 |
| 1. Family Medicine Groups/Network clinics | 1 | 2 | 3 | 4 | 9 |
| 1. Specialized medical clinics | 1 | 2 | 3 | 4 | 9 |
| 1. General and specialized hospitals that are not part of the CSSS | 1 | 2 | 3 | 4 | 9 |
| 1. University hospitals | 1 | 2 | 3 | 4 | 9 |
| 1. Community groups | 1 | 2 | 3 | 4 | 9 |
| 1. Pharmacies | 1 | 2 | 3 | 4 | 9 |
| 1. Health coops | 1 | 2 | 3 | 4 | 9 |

1. The following statements concern collaboration between your **CSSS** and the various **partners** identified in the previous question. Indicate the degree to which you agree or disagree with each statement.

|  | **Strongly**  **agree** | **Agree** | **Disagree** | **Strongly**  **disagree** | Don't know/ **Not applicable** |
| --- | --- | --- | --- | --- | --- |
| 1. It's easy to reconcile the values of the CSSS with those of local services network partners | 1 | 2 | 3 | 4 | 9 |
| 1. The CSSS's responsibilities and those of local services network partners related to service planning are clearly defined | 1 | 2 | 3 | 4 | 9 |
| 1. The CSSS plays an important role in the coordination of local services network | 1 | 2 | 3 | 4 | 9 |
| 1. Local network partners and CSSS professionals trust each other | 1 | 2 | 3 | 4 | 9 |
| 1. All partners are aware of the services offered by the CSSS | 1 | 2 | 3 | 4 | 9 |

1. Can you identify **your CSSS's three major partners in the local services network**? If there are fewer than three partners, write "none" in the remaining spaces.
2. ______________________________________________________________________
3. ______________________________________________________________________
4. ______________________________________________________________________
5. The following statements are about the effects of your CSSS's collaboration with various **partners in its territory**. Indicate the degree to which you agree or disagree with each statement.

| **Since the CSSS was created, collaboration between professionals at the CSSS and various partners in the territory has helped…** | **Strongly**  **agree** | **Agree** | **Disagree** | **Strongly**  **disagree** | Don't know/ **Not applicable** |
| --- | --- | --- | --- | --- | --- |
| 1. improve the territory clientele's access to primary care medical services | 1 | 2 | 3 | 4 | 9 |
| 1. improve the territory clientele's access to specialized care | 1 | 2 | 3 | 4 | 9 |
| 1. improve coordination of care among the various professionals in the territory | 1 | 2 | 3 | 4 | 9 |
| 1. improve clinical information sharing among the various professionals in the territory | 1 | 2 | 3 | 4 | 9 |
| 1. reduce service duplication (patient reassessments, repetition of diagnostic examinations, etc.) | 1 | 2 | 3 | 4 | 9 |
| 1. improve care management of vulnerable patients | 1 | 2 | 3 | 4 | 9 |
| 1. ensure greater continuity of care and services | 1 | 2 | 3 | 4 | 9 |
| 1. improve the quality of care and services | 1 | 2 | 3 | 4 | 9 |
| 1. gain better knowledge of their respective services | 1 | 2 | 3 | 4 | 9 |
| 1. prioritize needs and services to develop | 1 | 2 | 3 | 4 | 9 |

**5 - Support for the development of emerging primary care organization models**

1. Since your CSSS was created, to what degree have the following actions contributed to the development of **Family Medicine Groups (FMG)** and, if applicable, to **Network Clinics (NC)?**

|  | **A lot** | **Quite a lot** | **A little** | **Not at all** | **Was not implemented** |
| --- | --- | --- | --- | --- | --- |
| 1. Development of a common strategy with the regional general medicine directorate (DRMG) or its local representative | 1 | 2 | 3 | 4 | 99 |
| 1. Promotion of FMG/NC models by representatives of the CSSS or local DRMG | 1 | 2 | 3 | 4 | 99 |
| 1. Presentation of the pertinence of these models to various forums on primary care organization | 1 | 2 | 3 | 4 | 99 |
| 1. Creation of diagnostic and specialized service corridors for FMG/NC | 1 | 2 | 3 | 4 | 99 |
| 1. Offer of financial incentives and resources | 1 | 2 | 3 | 4 | 99 |
| 1. Support from the CSSS to the accredition process (for FMG/NC) | 1 | 2 | 3 | 4 | 99 |
| 1. Support for the development of nursing practice in primary care settings | 1 | 2 | 3 | 4 | 99 |
| 1. Support for the development of collective prescriptions | 1 | 2 | 3 | 4 | 99 |
| Others (specify): |  |  |  |  |  |
| _____________________________________  _____________________________________ | 1 | 2 | 3 | 4 | 99 |

1. Overall, to what degree has your CSSS supported and encouraged the development of **FMG** and, if applicable, of **NC** or **other emerging organizational models**?

1 A lot  2 Quite a lot  3 A little  4 Not at all

1. In your CSSS's territory, is there a **primary care organization that acts as a model** for the other clinics in the territory?

1 Yes 🢂 **Which one?**___________________________________________________________________________

2 No

1. To what degree has the **local DRMG representative** supported and encouraged implementation of **FGM** and **NC** in your CSSS's territory?

1 A lot _2_ Quite a lot _3_ A little _4_ Not at all

1. Would you say that **the medical clinics** in your CSSS's territory are **open to adopting new primary care organizational** **models**?

1 All _2_ Most _3_ A few _4_ None

**6 - Role of the Agence régionale and of the MSSS**

1. Think about the role of the **Agence régionale** in the development of your **CSSS** and **primary care services** in your territory. Indicate the degree to which you agree or disagree with each statement.

| **The Agence régionale …** | **Strongly**  **agree** | **Agree** | **Disagree** | **Strongly**  **disagree** | Don't know/ **Not applicable** |
| --- | --- | --- | --- | --- | --- |
| 1. supports your CSSS in the implementation of the local services network | 1 | 2 | 3 | 4 | 9 |
| 1. supports your CSSS in the development of service corridors with secondary and tertiary care institutions | 1 | 2 | 3 | 4 | 9 |
| 1. supports the activities of the local DRMG | 1 | 2 | 3 | 4 | 9 |
| 1. supports the development of primary medical care | 1 | 2 | 3 | 4 | 9 |
| 1. supports the emergence of new primary care services models (e.g. FMG/NC) | 1 | 2 | 3 | 4 | 9 |
| 1. Other roles (specify):   ____________________________________  ____________________________________ | 1 | 2 | 3 | 4 | 9 |

1. Think about the role of the **ministry of health (MSSS)** in the development of your **CSSS** and **primary care services** in your territory. Indicate the degree to which you agree or disagree with each statement.

| **The policies of the MSSS encourage…** | **Strongly**  **agree** | **Agree** | **Disagree** | **Strongly**  **disagree** | Don't know/ **Not applicable** |
| --- | --- | --- | --- | --- | --- |
| 1. the development of FMG/NC | 1 | 2 | 3 | 4 | 9 |
| 1. the development of primary care services | 1 | 2 | 3 | 4 | 9 |
| 1. the development of collaboration between primary care and other health system institutions | 1 | 2 | 3 | 4 | 9 |
| 1. the development of service corridors between primary, secondary and tertiary care institutions | 1 | 2 | 3 | 4 | 9 |
| 1. the development of local services networks | 1 | 2 | 3 | 4 | 9 |

**7 - Information about the respondent**

1. How long have you held your current position?

1 Less than a year _2_ 1 to 3 years _3_ 4 year or mroe

1. In what sector did you work **before you took this position**?

1 Hospital sector

2 Community sector (including CLSC)

3 Social services sector

4 Other sector? (specify): ____________________________________________________________________________

1. **How many years** have you been working in the health and social services sector?

1 Less than 5 years  2 5 to 15 years  3 More than 15 years

**THANK YOU FOR YOUR COLLABORATION!**

If you have any additional comments, please write them down in the space provided below.

We will be read them very carefully.

____________________________________________________________________________________________________

____________________________________________________________________________________________________

____________________________________________________________________________________________________

____________________________________________________________________________________________________

____________________________________________________________________________________________________

____________________________________________________________________________________________________

____________________________________________________________________________________________________

____________________________________________________________________________________________________

____________________________________________________________________________________________________

____________________________________________________________________________________________________

____________________________________________________________________________________________________

____________________________________________________________________________________________________

____________________________________________________________________________________________________

____________________________________________________________________________________________________

**Date: ________/_______/__________**

**(Day / Month / Year)**

April 2010

**PRINCIPAL INVESTIGATORS**

Jean-Frédéric Levesque

Raynald Pineault

Pierre Tousignant

**COLLABORATION**

Team of researchers and partners associated with this research project

**INSTUTIONAL SUPPORT**

(1) Canadian Institutes of Health Research (CIHR); (2) Fonds de recherche en santé du Québec (FRSQ); (3) Ministère de la Santé et des Services sociaux du Québec; (4) Agence de la santé et des services sociaux de Montréal – Direction de santé publique; (5) Agence de la santé et des services sociaux de la Montérégie; (6) Institut national de santé publique du Québec (INSPQ).

This project has been approved by the research ethics committee at the Agence de la santé et des services sociaux de Montréal.

Use of this questionnaire in whole or in part is not authorized without the consent of the study investigators.
