# Supplementary material for: Evaluation of the implementation of an integrated primary care network for prevention and management of cardiometabolic risk in Montréal
Source: BMC Fam Pract. 2011 Nov 10;12:126. doi: 10.1186/1471-2296-12-126 (PMC3282661; doi:10.1186/1471-2296-12-126)
Supplement: Additional file 9 — CSSS Questionnaire - End of project evaluation. This questionnaire, administered to key CSSS informants 40 months after project onset, will document the evolution of interorganizational collaboration within local services networks in relation to the program implementation. [file 1471-2296-12-126-S9.DOC]

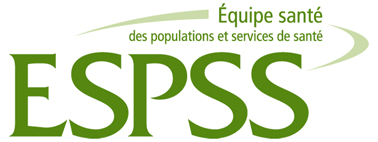
Additional file 9

**Evaluation of the implementation of an integrated primary care network for prevention and management of cardiometabolic risk in Montréal**

**CSSS Questionnaire**
**End of Project Evaluation**

**(draft version)**

April 2011


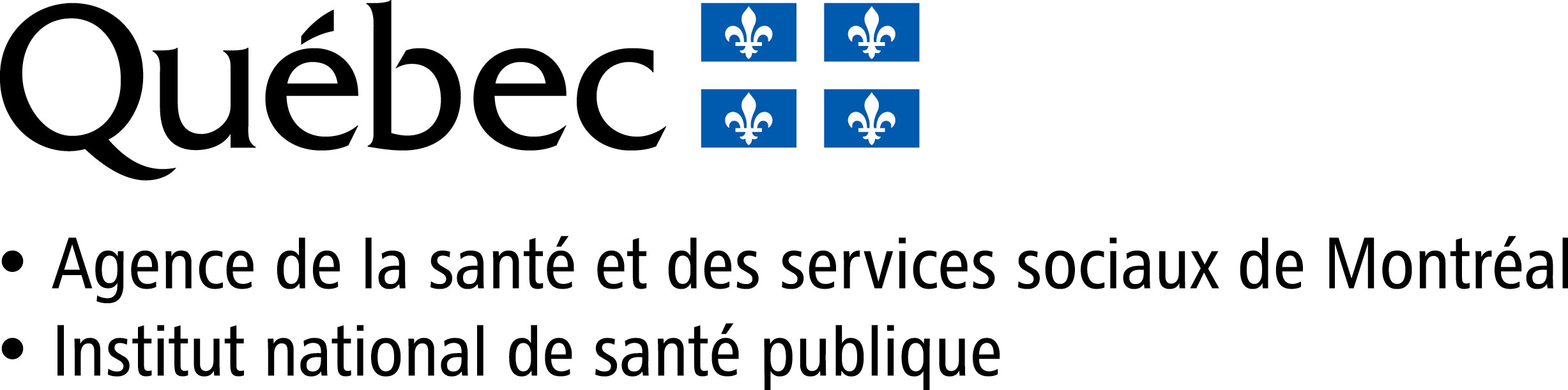


**Collaboration within the local services network**

1. The following statements concern the **impacts of implementing** the cardiometabolic risk program on collaboration between your CSSS and various partners in your territory. Please indicate the extent to which you agree or disagree with each of the following statements.

| **Implementing the program has helped …** | **Strongly**  **agree** | **Agree** | **Disagree** | **Strongly**  **disagree** | Don’t know/ **Does not apply** |
| --- | --- | --- | --- | --- | --- |
| 1. improve client access to primary care services in the territory |  |  |  |  |  |
| 1. foster better coordination of primary care services |  |  |  |  |  |
| 1. remove obstacles to collaboration between various levels of care |  |  |  |  |  |
| 1. encourage development of service corridors between primary and secondary care services |  |  |  |  |  |
| 1. improve client access to medical assessments or specialized care in the territory |  |  |  |  |  |
| 1. implement changes in professional practices |  |  |  |  |  |
| 1. improve coordination of care among various stakeholders in the territory |  |  |  |  |  |
| 1. improve clinical information sharing among various stakeholders in the territory |  |  |  |  |  |
| 1. reduce service duplication (e.g. patient reassessments, repetition of diagnostic exams) |  |  |  |  |  |
| 1. improve management of vulnerable patients |  |  |  |  |  |
| 1. ensure greater continuity of care and services |  |  |  |  |  |
| 1. improve quality of care and services |  |  |  |  |  |
| 1. develop better knowledge of services offered by various resources |  |  |  |  |  |
| 1. increase all beneficiaries' awareness of services offered in the territory |  |  |  |  |  |

1. Overall, to what degree has implementing the program helped develop collaborations

|  | **A lot** | **Quite a bit** | **Little** | **Not at all** | **Was not implemented** |
| --- | --- | --- | --- | --- | --- |
| 1. between your **CSSS (excluding the hospital)** and **primary care organizations** in your territory? |  |  |  |  |  |
| 1. between **primary care organizations** and **hospitals** in your territory (including the CSSS hospital, if applicable)? |  |  |  |  |  |
| 1. among **primary care organizations** in your territory? |  |  |  |  |  |

1. Do you totally or partly agree or partly or totally disagree with the following statements.

| **Overall, the cardiometabolic risk program** | **Strongly**  **agree** | **Agree** | **Disagree** | **Strongly**  **disagree** | Don’t know/ **Does not apply** |
| --- | --- | --- | --- | --- | --- |
| 1. has increased collaboration among the CSSS, primary care clinics and other resources of the local services network |  |  |  |  |  |
| 1. has consolidated the local services network |  |  |  |  |  |
| 1. has given rise to the creation of other chronic illness management networks |  |  |  |  |  |

1. At this time, how would you describe **collaboration between your CSSS** and the following partners?

|  | **Very good** | **Quite good** | **Poor** | **None** | Don’t know/ **Does not apply** |
| --- | --- | --- | --- | --- | --- |
| 1. Primary care clinics |  |  |  |  |  |
| 1. Family Medicine Groups/Network clinics |  |  |  |  |  |
| 1. Specialized medical clinics |  |  |  |  |  |
| 1. General and specialized hospitals that are not part of the CSSS |  |  |  |  |  |
| 1. University hospitals (RUIS) |  |  |  |  |  |
| 1. Community organizations |  |  |  |  |  |
| 1. Pharmacies |  |  |  |  |  |

1. The following statements refer to your **CSSS's collaboration with the various partners** listed in the previous question. Please indicate the extent to which you agree or disagree with each of the following statements.

|  | **Strongly**  **agree** | **Agree** | **Disagree** | **Strongly**  **disagree** | Don’t know/ **Does not apply** |
| --- | --- | --- | --- | --- | --- |
| 1. It is easy to reconcile the CSSS's values with those of partners in the local services network |  |  |  |  |  |
| 1. The CSSS's responsibilities regarding service planning and those of the partners in the local services network are clearly defined |  |  |  |  |  |
| 1. Local network partners and CSSS professionals trust each other |  |  |  |  |  |
| 1. All partners are aware of the services the CSSS offers |  |  |  |  |  |
| 1. The CSSS plays an important role in coordination of the local services network |  |  |  |  |  |

1. Can you identify your **CSSS's 3 major partners in the local services network**? Write "none" in the space if there are fewer than 3.
2. ______________________________________________________________________
3. ______________________________________________________________________
4. ______________________________________________________________________
5. In your CSSS territory, is there a **primary care organization that acts as a role model** for other clinics in the territory?

Yes 🢂 If yes, which one? __________________________________________________________________________

No

1. Would you say that the **medical clinics** in your CSSS's territory are **receptive** to adopting new primary care organizational models?

All of them  Most of them  A few of them  None of them

**Information about the person completing the questionnaire**

1. How long have you held your **current position?**

Less than a year  1 to 3 years  4 years or more

1. What is your current **position**?

Administrator in a CSSS

Head of local regional general medicine directorate (DRMG)

Other, *specify*: _______________________________________________________________________________________

1. **How many years** have you worked in the health and social services network?

Less than 5 years  5 to 15 years  More than 15 years

**THANK YOU FOR YOUR COOPERATION!**

If you have any additional comments, please write them down in the space provided below.

We will read them very attentively.

_______________________________________________________________________________________________________

_______________________________________________________________________________________________________

_______________________________________________________________________________________________________

_______________________________________________________________________________________________________

_______________________________________________________________________________________________________

_______________________________________________________________________________________________________

_______________________________________________________________________________________________________

_______________________________________________________________________________________________________

**Date: ________/_______/__________**

(Day / Month / Year)
